# Genetic rescue from protected areas is modulated by migration, hunting rate, and timing of harvest

**DOI:** 10.1111/eva.13554

**Published:** 2023-05-12

**Authors:** Roxane Lassis, Marco Festa‐Bianchet, Joanie Van de Walle, Fanie Pelletier

**Affiliations:** ^1^ Département de biologie et Centre d'Études Nordiques Université de Sherbrooke Sherbrooke Quebec Canada; ^2^ Biology Department Woods Hole Oceanographic Institution Woods Hole Massachusetts USA

**Keywords:** bighorn sheep, breeding migration, genetic rescue, individual‐based model, size‐selective harvest, source‐sink metapopulation dynamics

## Abstract

In terrestrial and marine ecosystems, migrants from protected areas may buffer the risk of harvest‐induced evolutionary changes in exploited populations that face strong selective harvest pressures. Understanding the mechanisms favoring genetic rescue through migration could help ensure evolutionarily sustainable harvest outside protected areas and conserve genetic diversity inside those areas. We developed a stochastic individual‐based metapopulation model to evaluate the potential for migration from protected areas to mitigate the evolutionary consequences of selective harvest. We parameterized the model with detailed data from individual monitoring of two populations of bighorn sheep subjected to trophy hunting. We tracked horn length through time in a large protected and a trophy‐hunted populations connected through male breeding migrations. We quantified and compared declines in horn length and rescue potential under various combinations of migration rate, hunting rate in hunted areas and temporal overlap in timing of harvest and migrations, which affects the migrants' survival and chances to breed within exploited areas. Our simulations suggest that the effects of size‐selective harvest on male horn length in hunted populations can be dampened or avoided if harvest pressure is low, migration rate is substantial, and migrants leaving protected areas have a low risk of being shot. Intense size‐selective harvest impacts the phenotypic and genetic diversity in horn length, and population structure through changes in proportions of large‐horned males, sex ratio and age structure. When hunting pressure is high and overlaps with male migrations, effects of selective removal also emerge in the protected population, so that instead of a genetic rescue of hunted populations, our model predicts undesirable effects inside protected areas. Our results stress the importance of a landscape approach to management, to promote genetic rescue from protected areas and limit ecological and evolutionary impacts of harvest on both harvested and protected populations.

## 1 INTRODUCTION

There are increasing concerns over the ecological and evolutionary changes induced by selective harvests of wild animals (Allendorf & Hard, 2009; Fenberg & Roy, 2008; Kuparinen & Festa‐Bianchet, 2017). In marine systems, long time series of fishery surveys, common garden and selection experiments (Kuparinen & Festa‐Bianchet, 2017; Sharpe & Hendry, 2009) supported the contention that by changing size‐dependent mortality, size‐selective fishing may drive changes in growth rate, timing of maturation, and reproductive investment in harvested species (Heino & Godø, 2002; Hutchings & Fraser, 2008). In terrestrial systems, harvest‐induced evolution has been explored by one study based on plant collections (Law & Salic, 2005) and a few long‐term studies of marked individuals or analyses of harvest records of mammals (Festa‐Bianchet, 2017; Kuparinen & Festa‐Bianchet, 2017). Evidence of harvest‐induced changes in life‐history traits of terrestrial species is limited (Gamelon et al., 2011; Kvalnes et al., 2016; Van de Walle et al., 2021; Zedrosser et al., 2011). Some ungulates selectively harvested based on the size or shape of their weapons (horns, antlers, or tusks) showed phenotypic changes consistent with hunting‐induced evolution (aoudad, *Ammotragus lervia* and Iberian ibex, *Capra pyrenaica*, Pérez et al., 2011; sable antelope, *Hippotragus niger*, Crosmary et al., 2013; African elephant, *Loxodonta africana*, Campbell‐Staton et al., 2021; Chiyo et al., 2015; bighorn sheep, *Ovis canadensis*, Pigeon et al., 2016; stone sheep, *Ovis dalli stonei*, Douhard et al., 2017; and European mouflon, *Ovis gmelini*, Garel et al., 2007). Other studies of hunted ungulates, however, documented no clear evidence of harvest‐induced evolutionary changes in weaponry, suggesting that an evolutionary response to selective hunting is only expected under very restrictive conditions (Büntgen et al., 2018; Festa‐Bianchet & Mysterud, 2018; Rughetti & Festa‐Bianchet, 2010).

Multiple ecological and management variables can modify the potential for evolutionary impacts of harvest, including the heritability of selected traits, as well as harvest intensity, selectivity, and its consistency over time and space (Festa‐Bianchet, 2017; Kuparinen & Festa‐Bianchet, 2017). As reservoirs of genetic and phenotypic diversity, and potential sources of migrants, protected areas can be part of landscape‐scale management to support sustainable harvest of wild species beyond their boundaries (Hansen, 2011; Kuparinen & Uusi‐Heikkilä, 2020; Watson et al., 2014). In theory, individuals that migrate from protected areas and breed in harvested populations could provide a genetic rescue (Tallmon et al., 2004; Whiteley et al., 2015) if gene flow through migration mitigated the evolutionary consequences of selective harvest (Baskett et al., 2005; Puckett & Eggleston, 2016; Tenhumberg et al., 2004).

Most empirical research on how protected areas could mitigate harvest‐induced evolution has focused on marine ecosystems (Hutchings & Fraser, 2008; Hutchings & Kuparinen, 2020). These studies suggest that marine protected areas can sometimes preserve or restore abundance, age structure and phenotypic trait distribution of exploited fish and invertebrate populations (Gillespie & Vincent, 2019; Sørdalen et al., 2020, 2022, synthesized by Lester et al., 2009; Baskett & Barnett, 2015). For instance, Russ et al., 2004 found that a no‐take reserve in the Philippines increased density and harvest of surgeonfish (*Acanthuridae* spp) and jacks (*Carangidae* spp) in nearby exploited areas. In terrestrial systems, a few studies investigated the potential of protected areas to limit the effects of trophy hunting on horn length of ungulates including impala, *Aepyceros melampus*, greater kudu, *Tragelaphus strepsiceros*, sable antelope, (Crosmary et al., 2013), and bighorn sheep (Lassis et al., 2022a; Poisson et al., 2020). These studies found limited evidence that protected areas provided a rescue to adjacent harvested areas.

Models of metapopulations with source‐sink dynamics have been widely used to assess the efficacy of marine reserves in providing rescue and to design reserve networks. These models can consider spatial changes in population density and account for multiple life‐history traits (Baskett et al., 2005; Dunlop et al., 2009; Puckett & Eggleston, 2016; Ratner & Lande, 2001). This approach, however, has been rarely applied to model source‐sink dynamics between protected and harvested areas in terrestrial systems, particularly for trophy‐hunted species (Tenhumberg et al., 2004). In a metapopulation, protected areas could be a source of unselected migrants, whereas harvested populations could act as sinks. The effectiveness of protected areas to buffer harvested populations against trait changes, however, depends on levels of harvest, reproduction, and migration (Hansen, 2011). In the absence of data on spatiotemporally explicit demographic rates within protected areas and migration rates between exploited and protected areas (Puckett & Eggleston, 2016), models of source‐sink dynamics could simulate realistic scenarios of demography, migration, and hunting rates to inform management seeking to favor genetic rescue. Empirical research and theory on metapopulation dynamics have explored how migration rates can affect size and persistence of different types of populations (Hansen, 2011; Hanski, 1998). Here, we are interested in whether a similar approach may inform managers about the age structure, numbers, and horn size of trophy‐hunted species when there are exchanges between protected and exploited populations.

In bighorn sheep, morphological changes consistent with hunting‐induced evolution of smaller horns have been reported only where males are subject to intense selective pressure through trophy hunting (Hengeveld & Festa‐Bianchet, 2011; Morrissey et al., 2021; Pigeon et al., 2016). Greater decreases in horn growth rate were documented in jurisdictions with higher harvest pressure on large‐horned males (LaSharr et al., 2019; Morrissey et al., 2021), supporting the crucial role of harvest rate on the probability that selective harvest would lead to evolutionary changes (Festa‐Bianchet, 2017). Although permanent dispersal in this species is rare (Festa‐Bianchet, 1986), many males undertake breeding migrations to seek mating opportunities outside their natal population (Hogg, 2000; Lassis et al., 2022a). Breeding migrants from protected areas could provide genetic contributions to harvested populations (Tallmon et al., 2004; Whiteley et al., 2015). In Alberta, Canada, however, the end of the hunting season overlaps with the beginning of breeding migration and some bighorn sheep males exiting protected areas are shot before they can mate (Lassis et al., 2022a). If most males leave protected areas before the hunting season ends, the potential for genetic rescue to dampen hunting‐induced selection on horn length will be weakened. Therefore, the efficiency of protected areas as a source of unselected individuals should vary over time and space depending on movement rates, demography, and both timing and intensity of selective harvest (Poisson et al., 2020; Tenhumberg et al., 2004). In addition, heavy harvest of males exiting protected areas could have undesirable selective effects for populations inside those protected areas. That is a particularly important concern in species such as bighorn sheep where surviving males return to their native population after the rut and may breed there in later years. Surprisingly, little modelling effort has been devoted to the possible consequences of harvest outside protected areas for populations inside protected areas.

The objective of this study was to assess to which extent male breeding migrations from protected areas can buffer selection for small horns in harvested populations, using parameters derived from long‐term studies and harvest records of bighorn sheep. We also explored how the intensity and timing of harvest could affect the potential for genetic rescue and the consequences of harvest on populations within harvest refuges. We tracked the distribution of horn length over time in a protected and a selectively hunted population using a stochastic individual‐based simulation (Grimm, 2018; Jørgensen & Fath, 2011). Although bighorn sheep in Canada form a metapopulation, with demographically distinct populations in both protected and hunted areas, we simulated a single exchange rate of males among one large hunted and one large, protected population, to reduce complexity. Individual‐based modelling is appropriate to simulate biological systems where individual variability is critical for population dynamics, age structure or trait distributions (Grimm, 1999; Grimm et al., 1999; Tenhumberg et al., 2004), With this flexible model, we simulated various male breeding migration rates and explored multiple scenarios of harvest regulations. Specifically, we tested three hypotheses concerning the most important factors affecting potential genetic rescue: migration rate, hunting rate in hunted areas, and timing of the harvest with respect to the timing of migrations. First, we expected a greater temporal decline in the average and standard deviation of horn length within the harvested population under high selective hunting rate (Hypothesis 1: Section 3.1). We expected a decline in standard deviation because hunting truncates the distribution of horn length in the population (Schindler et al., 2017). Second, we expected that as the rate of harvest of large‐horned males increased, the rate of breeding migration required for genetic rescue would also increase (Hypothesis 2: Section 3.2). Third, we expected that as the temporal overlap between hunting season and breeding migrations increased, the potential for migration to mitigate hunting‐induced effects on horn length would decrease (Hypothesis 3: Section 3.3). Although the direction of expected changes is obvious, their effect sizes are unknown, yet they are of crucial importance for management and conservation. Finally, we modelled the possible consequences of heavy harvest in hunted area for the morphological and genetic characteristics of populations inside protected areas.

## 2 MATERIALS AND METHODS

### 2.1 Model overview

We built an individual‐based model to track horn length of rams through time in a protected (*P*) and a harvested population (*H*). The model explicitly included breeding migrations; rams could migrate between populations before the rut, then return to their natal population after the rut. Notational conventions are summarized in Table S1.

At each annual time step, the model cycled over all individuals and computed horn growth, breeding, migration, and survival rates. The model considered four main annual processes that included several sub‐processes: (1) Development, including aging and horn growth, (2) Breeding, including yearling production, siring success, and offspring phenotype attribution, (3) Breeding migration, and (4) Survival, including both harvest and natural mortality. We ordered population sub‐processes within each time step to approximate the sequence of events over a bighorn sheep annual cycle (Figure S1.1a): aging and yearling production in spring; horn growth in summer; male breeding migration, harvest mortality and siring probability in autumn, and natural mortality over winter. We used 29 parameters (summarized in Table 1) to develop our model of the effects on these sub‐processes of individual characteristics such as age, sex, and horn length, and environmental variables such as population density and hunting rate. All processes, sub‐processes, and associated probabilities *p* at time *t* of each individual indexed by *i ∈* [0, *N*(*t*)], where *N*(*t*) is total population size, are described below.

**TABLE 1:** Parameters used in the individual‐based model to assess the potential for genetic rescue of horn length in bighorn sheep.

Description	Symbol	Equation	Values	Sources of parameters values
Horn growth				
Sex‐specific growth rates	$\rho_{f},\rho_{m}$	Equations 1 and 2	Table S2	Estimated
Sex‐specific asymptotic horn length	$y_{\infty ,f},y_{\infty ,m}$	Equations 1 and 2	Table S2	Estimated
Breeding				
Age‐ and density‐dependent yearling production by females	$\theta_{0},\theta_{1},\theta_{2},\theta_{3}$	Equation 3	Table S2	Estimated
Horn length‐dependent siring rates	$\;\gamma_{0},\gamma_{1}$	Equation 4	Table S2	Estimated
Offspring				
Recruitment sex ratio	σ		$\sigma =\frac{1}{2}$	Assumed equal
Intra‐ and inter‐sexual genetic variances for horn length	$V_{G_{\mathrm{ff}}},V_{G_{\mathrm{mm}}},V_{G_{\mathrm{fm}}}$	Equation 5	Table S3	Pigeon et al., 2016
Intra‐sexual environmental variances for horn length	$V_{E_{\mathrm{ff}}},V_{E_{\mathrm{mm}}}$	Equation 6	Table S3	Pigeon et al., 2016
Migration				
Permanent migration rate	$\varepsilon_{0}$		Table S3	Unpublished
Age‐specific and demography‐dependent breeding migration rates	$\lambda_{0},\lambda_{1},\lambda_{2},\lambda_{3},\lambda_{4}$	Equation 7	Table S3	Lassis et al., 2022a
Flow scale	ε	Equation 7	Table S5	Explored
Harvest				
Minimum legal horn length	$y_{\min}$	Equation 9	Figure S1.3	Estimated
Hunting rate	φ	Equation 9	Table S5	Explored
Timing overlap with migrations	ω		Table S5	Explored
Natural mortality				
Age‐ and sex‐specific mortality rates	$\mu_{0},\mu_{1},\mu_{2}$	Equation 10	Table S5	Loison et al., 1999

*Note*: Parameter values were either estimated through Bayesian regressions of long‐term data from monitoring individually marked bighorn sheep in Ram Mountain and Sheep River populations (“Estimated”), derived from the literature, explored to simulate multiple scenarios (“Explored”), or based on assumptions to limit model complexity (“Assumed”). Reference to the equation in which each parameter is involved as well as Tables where each parameter value can be found are also provided.

#### 2.1.1 Development

##### Aging

At every time step, each individual *i* ages by 1 year. Both sexes become sexually mature between 2 and 4 years of age (Ritchot et al., 2021), consequently participation in yearling production for females and siring competition for males started at 2 years of age.

##### Horn growth

Between time *t* and *t* + 1, horn length increases by an increment Δy. At time *t*, the horn length of individual *i* of age $a_{i}$ and sex $s_{i}$ was defined by the von Bertalanffy growth equation (Mccallum, 2008):

(1) $$y_{i}\left(a_{i},s_{i}\right)=y_{\infty {,}_{i}}\left(1-e^{-\rho \left(s_{i}\right)a_{i}}\right)$$

where $y_{\infty ,i}$ is the asymptotic horn length of individual *i* and $\rho \in \rho_{f},\rho_{m}$ are sex‐specific horn growth rates (Table S2, Figure S1.2a,b). Then from Equation 2, the horn growth increment, Δy, of an individual *i* of age $a_{i}$ and sex $s_{i}$ between time *t* and *t* + 1 was:

(2) $$\mathrm{\Delta y}=\left(y_{\infty {,}_{i}}-y_{i}\left(a_{i},s_{i},t\right)\right)\left(1-e^{-\rho \left(s_{i}\right)}\right)$$

#### 2.1.2 Breeding

##### Yearling production

At every time step *t*, a female *i* could produce an offspring that will recruit in the population as a yearling at *t* + 1. Bighorn ewes conceive only one lamb per year (Festa‐Bianchet et al., 2019). We used yearling production to avoid the complexity of modelling the high annual variability in lamb mortality (Feder et al., 2008; Festa‐Bianchet et al., 2019). Yearling production rate was thus the product of lamb production and lamb survival to 1 year, accounting for maternal age and density effects. At time *t*, the probability that a female *i* of age $a_{i}$ and population of origin $o_{i}$ recruited a yearling at time *t* + 1, was:

(3) $$p_{\text{recruit}}\left(a_{i},o_{i},t\right)=\frac{1}{1+\exp\left(-\left(\theta_{0}+\theta_{1}a_{i}+\theta_{2}a_{i}^{2}+\theta_{3}N_{o}\left(o_{i},t\right)\right)\right)}$$

$\text{with}\;\theta_{0},\theta_{1},\theta_{2},\theta_{3}$ being the regression coefficients of ewe age and population size on recruitment rate (estimates in Table S2, Figure S1.2c,d) and $N_{o}\in N_{P},N_{H}$ the total number of individuals of both sexes within the population of origin.

##### Siring success

Male siring success was dependent upon horn length in simulations. Horn length is correlated with social rank (Martin et al., 2013), a major determinant of male reproductive success (Coltman et al., 2002; Hogg & Forbes, 1997; Pelletier & Festa‐Bianchet, 2006). Bighorn sheep are polygynous and male mating competition largely depends on horn length (Geist, 1971; Hogg & Forbes, 1997). During the rut, dominant males use a tending tactic to defend one estrous females at a time, while subordinate males resort to alternative tactics such as coursing to attempt to copulate with tended females (Hogg, 1984, 1987; Hogg & Forbes, 1997). Tending is the most efficient tactic, leading to a skewed distribution of paternities in favor of the top‐ranking and largest‐horned mature males, but 40% of lambs are sired by subordinates (Coltman et al., 2002). Siring success is likely independent of demography for older and dominant males but for younger and subordinate males it decreases as the number of competitors increases (Martin et al., 2016; Ritchot et al., 2021). Consequently, we simulated siring competition among adult males according to their horn length. The siring probability of a male *i* of horn length $y_{i}$ at a time *t* was defined as:

(4) $$p_{\text{siring}}\left(y_{i},t\right)=\frac{1}{1+\exp\left(-\left(\gamma_{0}+\gamma_{1}y_{i}\right)\right)}$$

where $\gamma_{0}\;\text{and}\;\gamma_{1}$ are regression parameters linking horn length and siring probability (Table S2, Figure S1.2e).

We assigned the paternity of each yearling recruit in a population $o_{i}$ at a time *t* + 1 according to the weighted siring chances $p_{\text{siring}}$ of males within the same population $o_{i}$ during the breeding season at time *t* − 1. That is because a surviving lamb conceived during the rut in year *t* − 1 and born in year *t* would be recruited as a yearling in year *t* + 1.

##### Offspring phenotype attribution

Sex, $s_{i}$, and asymptotic horn length, $y_{\infty ,i}$, were allocated to each yearling assuming equal sex ratio at recruitment. Long‐term studies of pedigreed bighorn populations revealed that horn length has a strong genetic component and estimated its intra‐ and inter‐sexual genetic and environmental variances (Coltman et al., 2005; Miller et al., 2018; Pigeon et al., 2016; Poissant et al., 2008). These estimates allow to compute breeding values that quantify the expected deviation of an individual's horn length from the population mean attributable to the additive genetic component (Wilson et al., 2016). Because bighorn sheep are diploid and horn length is sexually dimorphic, we attributed to each individual of both sexes a breeding value for both female, $x_{f,i},$ and male, $x_{m,i},$ asymptotic horn length. The breeding value of yearling i for asymptotic horn length, $x_{i}=\left(x_{f,i}\;x_{m,i}\right)$, was calculated from the breeding values of its mother $i^{\prime },x_{i^{\prime }}=(\begin{array}{cc} x_{f,i^{\prime }} & x_{m,i^{\prime }} \end{array})$, and father $i^{\prime \prime },x_{i^{\prime \prime }}=(\begin{array}{cc} x_{f,i^{\prime \prime }} & x_{m,i^{\prime \prime }} \end{array})$, as:

(5) $$x_{i}=N\left(\frac{1}{2}\left(x_{i^{\prime }}+x_{i^{\prime \prime }}\right),,,V_{G}\right)$$

where N refers to a multivariate normal distribution with a mean corresponding to the average parental breeding values for sex‐specific asymptotic horn length and a variance $V_{G}=\left(\begin{array}{cc} V_{G_{\mathrm{ff}}} & V_{G_{\mathrm{fm}}}\\ V_{G_{\mathrm{fm}}} & V_{G_{\mathrm{mm}}} \end{array}\right)$ corresponding to the intra‐ and inter‐sexual genetic variances for horn length estimates from literature (Table S3).

The asymptotic horn length of a yearling *i* of sex $s_{i}$ and horn length breeding value $x_{i}$ was computed as:

(6) $$\begin{array}{c} y_{\infty ,i}\left(x_{i},s_{i}\right)=\left\{\begin{array}{cc} N\left(\bar{y_{\infty ,f}}+x_{f,i},\sqrt{V_{E_{f}}}\right) & \text{if}\;s_{i}=F\\ N\left(\bar{y_{\infty ,m}}+x_{m,i},\sqrt{V_{E_{m}}}\right) & \text{if}\;s_{i}=M \end{array}\right. \end{array}$$

where N is a normal distribution with a mean corresponding to the sum of yearling breeding values $x_{i}$ and the population average asymptotic horn lengths for females or males with $\bar{y_{\infty ,f}}=\frac{1}{N_{f}}\sum_{j=1}^{N_{f}}y_{\infty ,j}$ and $\bar{y_{\infty ,m}}=\frac{1}{N_{m}}\sum_{j=1}^{N_{m}}y_{\infty ,j}$ (Falconer & Mackay, 1996); with a standard deviation $V_{E}=\left(\begin{array}{cc} V_{E_{f}} & V_{E_{m}} \end{array}\right)$ corresponding to the environmental variances of horn length for females and males (literature estimates in Table S3).

#### 2.1.3 Breeding migration

Permanent dispersal of bighorn sheep is rare (Festa‐Bianchet, 1986). Consequently, we only modelled gene flow between protected and harvested populations through male breeding migrations. Information on determinants, timing, and rates of male breeding migrations comes from the long‐term study of bighorn sheep at Sheep River, Alberta (Lassis et al., 2022a), that found that the probability of breeding migration increases for middle‐aged males, increases with the number of adult males, and decreases with female/male adult sex ratio during the pre‐rut. Migration rates were implemented in our model based on observations at Sheep River, a small, protected area surrounded by hunted areas. To make our model more general, we added a parameter ε, termed “flow scale” to allow breeding migration to vary in time and space according to landscape connectivity. By varying the flow scale, we simulated conservative scenarios where the probability of breeding migration was lower than observed at Sheep River. In other words, *ε* represents a fraction of migration rates calculated from Sheep River data, with higher values corresponding to higher migration rates (Figure S3.1).

Each year, males could undertake breeding migrations. The probability of breeding migration depended upon mating opportunities within the population of origin, determined by male age and local availability of reproductive females (Hogg & Forbes, 1997; Lassis et al., 2022a). Therefore, the probability of male *i* of age $a_{i}$ and population of origin $o_{i}$ to migrate temporally into the other population between times *t* and *t* + 1 was derived from Lassis et al. (2022a) as:

(7) $$p_{\text{migration}}\left(a_{i},o_{i},t\right)=\varepsilon \frac{1}{1+\exp\left(-\left(\lambda_{0}+\lambda_{1}a_{i}+\lambda_{2}a_{i}^{2}+\lambda_{3}\mathrm{sr}\left(o_{i},t\right)+\lambda_{4}N_{o,m}\left(o_{i},t\right)\right)\right)}$$

where $\varepsilon \in \left[0,1\right]$ is the flow scale between populations (values in Tables S5 and S6). Parameters $\lambda_{0},\lambda_{1},\lambda_{2},\lambda_{3},\lambda_{4}$ are the regression coefficients of age and demographic characteristics on male breeding migration rate (literature estimates in Table S3); sr the adult sex ratio (Equation 8); and $N_{o,m}\in N_{P,m},N_{H,m}$ the number of competing males in the population of origin.

(8) $$\mathrm{sr}\left(o_{i},t\right)=\left\{\begin{array}{cc} \frac{N_{P,f}\left(t\right)}{N_{P,m}\left(t\right)} & \text{if}\;o_{i}=P\\ \frac{N_{H,f}\left(t\right)}{N_{H,m}\left(t\right)} & \text{if}\;o_{i}=H \end{array}\right.$$

where $N_{P,f},$ $N_{H,f},$ $N_{P,m}$, and $N_{H,m}$ are the numbers of females (*f*) and males (*m*) in the population of origin.

#### 2.1.4 Survival

##### Harvest mortality

Hunting regulations are often based on horn length thresholds and/or quotas to limit the harvest (Festa‐Bianchet, 2017; Festa‐Bianchet & Mysterud, 2018). We used the quota‐free 4/5‐curl regulation prevalent in most of Alberta over the past 45 years (Festa‐Bianchet et al., 2014) which defines as “legal” for hunting a bighorn male whose horns describe at least 4/5 of a complete curl, mostly determined by horn length (see Figure 1 in Pelletier et al., 2012). At every time step *t*, the probability of being harvested for a male *i* of horn length $y_{i}$ within the harvested population ($o_{i}=H$) was determined according to a minimum horn length rule as:

(9) $$p_{\text{harvest}}\left(y_{i},t\right)=\left\{\begin{array}{cc} \varphi & \text{if}\;y_{i}\geq y_{\min}\;\\ 0 & \text{otherwise} \end{array}\right.$$

where φ is the hunting rate of “legal” males (values in Tables S5 and S6); and $y_{\min}$ is the minimum legal horn length, which was set at 76.8 cm (Figure S1.3).

**FIGURE 1:**
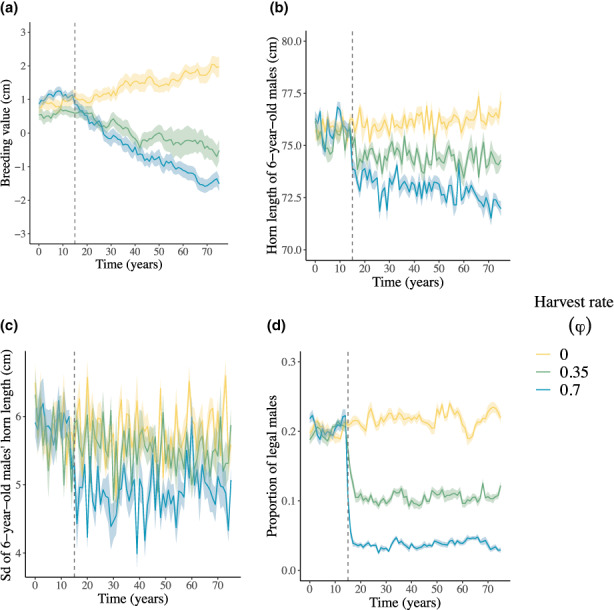
Effects of hunting rate (*φ*, with flow scale *ε* = 0.50 and timing of harvest overlap with migrations *ω* = 0.35) on the annual (a) mean breeding value for male horn length (cm), (b) mean horn length of 6‐year‐old males (cm), (c) standard deviation of horn length of 6‐year‐old males (cm), and (d) mean proportion of legal males in the harvested population. Lines and shades represent the averages, and the associated 95% confidence intervals over all replicate runs. The vertical dashed line marks the start of annual harvest at *T*_*H*_ = 15 years.

Furthermore, to vary the temporal overlap between the hunting season and breeding migrations, we included the additional parameter ω, which sets the percentage of migrations occurring while hunting was allowed (Tables S5 and S6).

##### Natural mortality

Bighorn sheep survival varies with sex and age (Loison et al., 1999). As we simulated recruitment of yearlings, lamb survival was indirectly accounted for through yearling production (Equation 3, estimates in Table S2, Figure S1.2c,d). At every time *t*, the probability that an individual *i* of age $a_{i}$ and sex $s_{i}$ died of natural causes was derived from the Bernoulli distribution with a logit link function as follows:

(10) $$p_{\text{dying}}\left(a_{i},s_{i}\right)=1-\frac{1}{1+\exp\left(-\left(\mu_{0}\left(s_{i}\right)+\mu_{1}\left(s_{i}\right)\times a_{i}+\mu_{2}\left(s_{i}\right)\times {a_{i}}^{2}\right)\right)}$$

where $\mu_{0},\mu_{1},\mu_{2}$ are the sex‐specific effects of age on natural mortality rate (literature estimates in Table S3).

### 2.2 Simulation design

#### 2.2.1 Parametrization

We based estimation of model parameters (Tables S2 and S3) and initial conditions (Table S4) on the Ram Mountain and Sheep River populations of bighorn sheep in Alberta, Canada, with detailed knowledge of life history and population dynamics from decades of monitoring marked individuals (Festa‐Bianchet et al., 2019). The province of Alberta includes protected and hunted areas. In the latter, there is a high harvest pressure on “legal” rams, which likely varies from 30 to 70% depending on the area (Festa‐Bianchet et al., 2014; Pelletier et al., 2014; Poisson et al., 2020).

#### 2.2.2 Simulations

We first ran the model for 25 years without hunting to allow age and horn length distributions to attain equilibria. We then ran the model over 75 additional years. We presented results upon equilibrium, which we considered as the new initial time (*T*_*I*_ = 0 year). Year 75 post‐equilibrium was the final year of simulation, *T*_*F*_ = 75 years (Figure S1.1b). Hunting started 15 years after equilibrium at *T*_*H*_ = 15 years. To test our hypotheses, we varied harvest pressure through changes in hunting rates of “legal” rams (Hypothesis 1: Section 3.1), we varied flow scale between populations along with hunting rate (Hypothesis 2: Section 3.2), and we varied the temporal overlap of hunting season and breeding migrations along with hunting rate (Hypothesis 3: Section 3.3). All simulated scenarios are presented in Tables S5 and S6.

For each scenario, we executed 10 replicate runs with the same parameterization that started with the same initial conditions and varied only through the stochasticity of life‐history and demographic processes. We presented temporal trajectories over 75 years of yearly mean breeding value for male horn length in each population, as well as age‐specific horn length. We chose this metric rather than population age structure to avoid bias from harvest‐induced changes in population age structure. As hunting targets males from the age of 4 years and on average at 7 years (Festa‐Bianchet et al., 2014), we reported temporal trends of the average and standard deviation of horn length of 6‐year‐olds. We also presented the temporal trends in proportion of legal males, that are available for hunting.

Over 60 years of harvest, we measured temporal decline in breeding value, male horn length and proportion of legal males as the differences between the average final value at *T*_*F*_ and the average value at *T*_*H*_ over all simulation runs of each scenario. We quantified rescue potential as the attenuation of decline in breeding value for male horn length compared to a scenario without migration. We computed the frequency of decline attenuations as the percentage of simulation runs ending with a mean breeding value for male horn length higher than the upper 95% confidence interval of breeding value obtained without migration at *T*_*F*_.

#### 2.2.3 Sensitivity analysis

We tested the sensitivity of model predictions to changes in life‐history parameters affecting horn growth, horn length heritability and sexual selection for horn length. For each parameter separately, we ran 50 replicate runs with the parameter set to its mean value perturbed by ±5%, holding other parameters at their mean value (Jørgensen, 1986). We compared the mean decline in breeding value for male horn length with the perturbed parametrization, $D_{\text{sens},\pm 5\%}$, with the mean decline obtained under a reference scenario where all parameters were fixed to their mean value, $D_{\mathrm{ref}}$, over 60 years of harvest. We also reported the sensitivity value, $S_{\pm 5\%}$, defined as the ratio between the computed change in the mean decline of breeding value in male horn size and the applied perturbation in the particular parameter's value (Table S8).

## 3 RESULTS

### 3.1 Hypothesis 1: Effect of legal ram hunting rate

Selective hunting of males led to a decline in breeding value for male horn length, and consequently in the age‐specific mean and standard deviation of male horn length, along with the proportion of legal males in both populations. Declines were slower in the protected than in the hunted population and there was no decline in the protected population in the absence of migration (Figures S2.1 and S2.2).

In the hunted population, with intermediate values for flow scale (ε = 0.50) and temporal overlap of hunting and migrations (ω = 0.35), horn length breeding value decreased by 1.03 and 2.62 cm on average over all simulation runs with 35% and 70% hunting rates, respectively (Figure 1a). Under 35% and 70% hunting rates, horn length of 6‐year‐old males decreased by 0.91 and 3.50 cm (Figure 1b), its standard deviation was constant and decreased by 0.74 cm (Figure 1c), and the proportion of legal males decreased by 8.3% and 16.9% (Figure 1d), respectively, during these simulations.

### 3.2 Hypothesis 2: Interplay between legal ram hunting rate and flow scale

All these simulations kept the timing overlap between hunting and migrations at an intermediate value, ω = 035. The annual migration rate increased with the flow scale (ε) and decreased with increasing hunting rate (*φ*, Figure S3.1), because when mortality of large males increases, local competition decreases and so does the probability of breeding migration. This effect was observed in both harvested and protected populations when timing of harvest overlapped with migrations.

**FIGURE 2:**
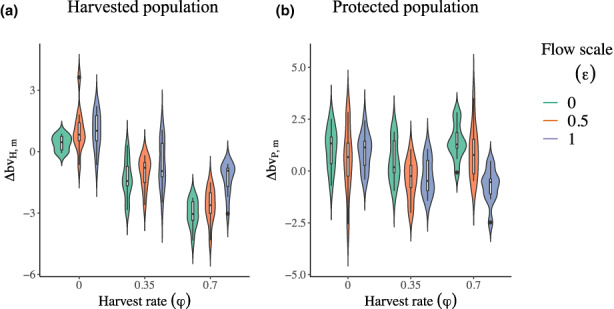
Combined effects of hunting rate (*φ*, with timing of harvest overlap with migrations *ω* = 0.35) and flow scale (*ε*) on changes over 60 years of harvest in mean breeding value for horn length of bighorn sheep males (∆*bv*_*m*_, differences in cm between the average value at *T*_*F*_ = 75 years and before *T*_*H*_ = 15 years) in the (a) harvested and (b) protected populations. The black line in the box indicates the median, the lower and upper edges mark the 1st and 3rd quartiles, and the lower and upper whiskers extend to the smallest and largest values no further than 1.5 times the inter‐quartile range over all replicate runs. Violin shapes represent density estimates of the *y*‐axis variables in each combination of values for *φ* and *ε*.

**FIGURE 3:**
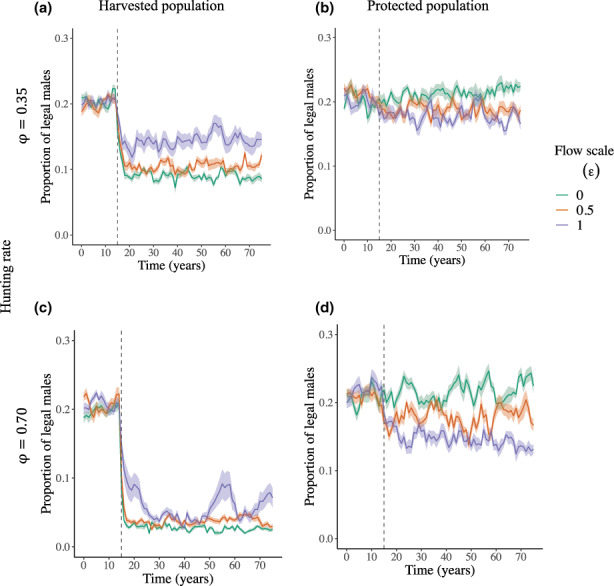
Effects of flow scale (*ε*) under different hunting rates (*φ* = 0.37 or 0.70, with timing of harvest overlap with migrations *ω* = 0.35) on the annual proportion of legal males in harvested (a, c) and protected (b, d) populations. Lines and shades represent averages and associated 95% confidence intervals over all replicate runs. The vertical dashed line marks the start of annual harvest at *T*_*H*_ = 15 years.

In the hunted population, the decline in breeding value and age‐specific mean of male horn length were greater at low flow scale (Figure 2, Figures S3.2 and S3.3). For instance, over 60 years of high hunting rate (φ = 0.70), horn length breeding value declined by 2.62 cm and 1.32 cm with flow scales (ε) of 0.50 and 1.00, respectively (Figure 2a, Figure S3.2c). The rescue potential for breeding value for male horn length increased with the flow scale (ε), particularly at high hunting rates (φ, Figure 2a). With low hunting rate φ = 0.35, a rescue potential after 60 years of harvest was detected in 30% and 40% of simulation runs with flow scales (ε) of 0.50 and 1.00, respectively (Figure 2a, Figure S3.2a). With high hunting rate (φ = 0.70), however, a rescue potential was detected in 70% and 90% of simulation runs with flow scales (ε) of 0.50 and 1.00 respectively (Figure 2a, Figure S3.2c). Across all simulation runs at high hunting rate (φ = 0.70), the proportion of legal males dropped by 16.9% and 14.1% with a flow scale (ε) of 0.50 and 1.00, respectively (Figure 3c).

In the protected population, the final means of breeding value for male horn length decreased with increasing flow scale (ε), particularly at high hunting rate (Figure 2, Figures S3.2 and S3.3). With high hunting rate (φ = 0.70) and flow scale (ε = 1.00), average breeding value declined by 0.72 cm (Figure 2b, Figure S3.2d). Declines in proportion of legal males (Figure 3), proportion of older individuals and male: female ratio (Figures S3.4 and S3.5) were larger with increasing flow scale (ε). For instance, at high hunting rate (φ = 0.70), the proportion of legal males dropped by 3.78% and 7.73% with flow scales (ε) of 0.50 and 1.00 respectively (Figure 3d).

**FIGURE 4:**
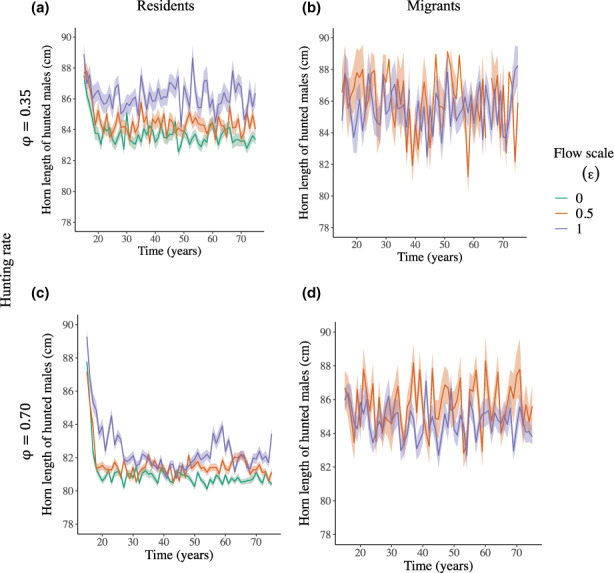
Effects (from harvest start at *T*_*H*_ = 15 years) of flow scale (*ε*) under different hunting rates (*φ* = 0.35 or 0.70, with timing of harvest overlap with migrations *ω* = 0.35) on the annual mean of male horn length (cm) of hunted bighorn sheep males according to their origin (a, c: residents from the harvested population; or b, d: migrants from the protected population). Lines and shades represent the averages and the associated 95% confidence interval between all replicate runs.

Considering harvested males only, the effects of breeding migrations on the evolutionary trends in horn length became more pronounced as the hunting rate (φ) increased and were dampened by more migrants in the harvest as the flow scale (ε) increased (Figure 4, Figure S3.6). The temporal decline in horn length breeding value and horn length of hunted males originating from the hunted population increased with increasing hunting rate (φ) and decreasing flow scale (ε), but it increased with hunting rate at any flow scale (Figure 4).

### 3.3 Hypothesis 3: Interplay between hunting rate and overlap of harvest with breeding migrations

All these simulations used an intermediate flow scale, ε = 0.50. A greater overlap of the hunting season with male breeding migrations (ω) increased the proportion of the harvest that was made up of males originating from the protected area (Figure S4.1). Consequently, the breeding value for male horn length, age‐specific male horn length and proportion of legal males in the protected population decreased at high hunting rate (φ = 0.70, Figure S4.2).

**FIGURE 5:**
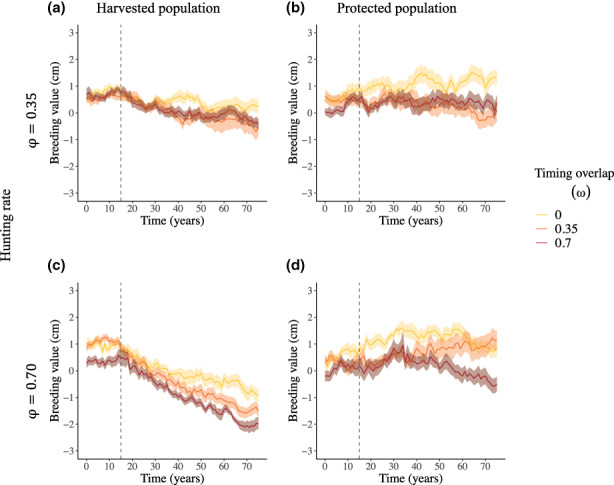
Effects of timing of harvest overlap with migrations (*ω* = 0.00, 0.35 or 0.70) under different hunting rates (*φ* = 0.35 or 0.70, with flow scale *ε* = 0.50) on the annual mean breeding value for male horn length (cm) within harvested (a, c) and protected (b, d) populations. Lines and shades represent averages and associated 95% confidence intervals of all replicate runs. The vertical dashed line marks the start of annual harvest at *T*_*H*_ = 15 years.

In the harvested population, at low hunting rate (φ = 0.35), the temporal trajectories of horn length breeding value were almost flat, with a positive effect of no overlap of harvest and migrations (Figure 5a). At high hunting rate (φ = 0.70), however, the decline in breeding value of male horn length increased with greater overlap of harvest and migrations (Figure 5c, Figure S4.2a). With high hunting rate (φ = 0.70), a rescue potential after 60 years of harvest was detected in 80%, 70% and 40% of simulation runs with overlap (ω) of 0.00, 0.35, and 0.70 respectively.

A decline in breeding value of male horn length was evident also in the protected population when hunting rate and overlap with breeding migrations were high (ω = 0.70 and φ = 0.70), with a decrease of 0.70 cm on average over all simulation runs (Figure 5d, Figure S4.2b).

## 4 DISCUSSION

Our model produced two key results that are relevant to manage a mosaic of selectively hunted and protected populations over a landscape. First, breeding migration from protected to hunted populations can dampen phenotypic and genetic effects of selective harvests. Second, when the timing of breeding migration coincides with intense selective harvest, rather than a genetic rescue of harvested populations our model predicts undesirable evolutionary changes within both harvested and protected areas.

For hunted populations, our model predicted a decline of about 3.50 cm in age‐specific horn length and 2.62 cm in breeding value for male horn length over 60 years, or about 8.5 bighorn sheep generations, under strong harvest pressure, intermediate level of migration between the protected and hunted areas and intermediate timing overlap of hunting with migration. Our model simulations quantified harvest‐induced declines in both the population average and standard deviation of horn length over time. For 6‐year‐olds, the decline in standard deviation for horn length can be explained by the selective removal of males with longer horns. At the population level when considering all male age classes, we observed an even greater decline in standard deviation of horn length, because hunting truncates population age structure and horn length distribution. Impacts of harvest on population dynamics through modifications in proportion of legal males, age structure and sex ratio should concern managers. These demographic changes not only influence the long‐term availability of trophy males, and consequently harvest opportunities, but could also have indirect detrimental effects on population growth (Aspbury et al., 2019; Deakin et al., 2022; Milner et al., 2007; Pelletier et al., 2007; Van de Walle et al., 2021).

As expected, our results show a higher potential for genetic rescue with low hunting rates, high migration rates, and protection of migrants from harvest. The risk of evolutionary decline in male horn size could be limited by reducing harvest rate (Kuparinen & Festa‐Bianchet, 2017). Long‐term monitoring of harvested mountain sheep reported stable temporal trends in horn length under low hunting pressure and declines in horn length under intense hunting pressure (Stone sheep in British Columbia: Douhard et al., 2016; bighorn sheep in British Columbia: Hengeveld & Festa‐Bianchet, 2011; multiple jurisdictions in Canada and United States: LaSharr et al., 2019; Morrissey et al., 2021). Although the harvest rate of legal males can rarely be estimated, because the availability of harvestable males is unknown (Festa‐Bianchet & Mysterud, 2018), available estimates indicate that it can be very high in some jurisdictions (about 40%–67% in Alberta, Coltman et al., 2003; Parks Canada, 2020; Pigeon et al., 2016). Our simulations suggest that at high hunting rates genetic rescue of harvested populations is weak even with high rates of breeding migration from protected areas. Instead, high hunting rates lead to harvest‐induced declines in breeding value of male horn size also in the protected population, especially with high flow scale or temporal overlap of harvest with migration.

The harvest of migrant males before the rut limits the potential for genetic rescue to buffer artificial selection in harvested populations. Closing the hunting season before breeding migrations start in mid‐October (Lassis et al., 2022a) would increase the probability of successful mating and the genetic contribution to harvested populations of migrants from protected areas (Pelletier et al., 2012; Poisson et al., 2020). An earlier end to the hunting season would also avoid the artificial effects we found for populations inside protected areas. Breeding migration partially removes the protection that National Parks and other protected areas provide to populations within their boundaries. We modelled migration probability based on local conditions, notably the level of competition among males and the adult sex ratio (Lassis et al., 2022b). However, bighorn males also conduct prospective trips in October–November to assess their breeding potential in other populations (Lassis et al., 2022a). High hunting pressure, by reducing the number of large and competitive males, may increase the incentive of males to migrate from protected to harvested areas (Hogg, 2000). As local horn length declines, an increasing proportion of males would not be sampled through the legal hunt because they do not fit the legal definition of minimum curl (Pelletier et al., 2012), and an unknown but likely increasing proportion of migrants from neighboring parks would be shot (Lassis et al., 2022b; Pelletier et al., 2014; Poisson et al., 2020), reducing the reliability of harvest data to monitor local temporal trends in horn size (Festa‐Bianchet et al., 2015; Leclerc et al., 2016; Pelletier et al., 2012). Notably, we found that at high harvest rate, an increasing proportion of the harvest is made up of males originating from protected areas.

We parameterized life‐history and demographic processes in our model based on empirical estimates of the genetic and phenotypic components of horn length from long‐term studies of two pedigreed bighorn populations in Alberta (Coltman et al., 2005; Miller et al., 2018; Pigeon et al., 2016; Poissant et al., 2008), along with the role of horn length in male reproductive success (Hogg & Forbes, 1997; Pelletier & Festa‐Bianchet, 2006). Although we did not assess their relative effects, we used a probabilistic approach to indirectly take into account other environmental drivers such as weather, habitat quality or population density that also affect horn growth (Douhard et al., 2017; Festa‐Bianchet et al., 2004). We confirmed the robustness of the predicted temporal decline in horn length breeding value over 60 years with multiple replicate runs (Table S7) and tested the sensitivity of our model predictions to changes in values of parameters quantifying horn growth, horn length heritability, and sexual selection for horn length (Table S8). Higher sensitivities were observed to perturbations in male asymptotic horn length and the regression parameters linking male horn length and siring probability, while the model prediction were less sensitivities to changes in heritability of female horn length.

Male horn length in unhunted populations is likely under stabilizing selection, but we had no empirical basis to estimate selection against very large horns in the absence of trophy hunting. Consequently, our model included a positive natural selection on horn length through male mating success, and a negative artificial selection through hunting. That modelling choice led to temporally increasing breeding values for male horn length in the absence of harvest (Table S7), reducing the simulated effects of size‐selective harvest compared to a real‐world situation where breeding value, in the absence of hunting, is presumably at equilibrium. Consequently, our model predictions likely underestimate temporal declines in horn length breeding value and male horn length within both harvested and protected populations. For instance, Festa‐Bianchet et al. (2014) reported a decrease of about 2.6 cm of age‐specific horn length over 4.3 generations in bighorn males in Alberta. The only genetic evidence for hunting‐induced evolutionary change in bighorn sheep, from long‐term research at Ram Mountain, Alberta, documented that 17% of the overall phenotypic decrease in horn length, corresponding to about 2.6 cm over 3.3 generations, was due to evolutionary change under a 4/5‐curl regulation (Pigeon et al., 2016).

Individual‐based models incorporating source‐sink metapopulation dynamics can guide harvest regulations at the landscape scale (Hansen, 2011; Puckett & Eggleston, 2016). Using this approach, we tested three hypotheses about how a metapopulation structure, with exchanges of males between hunted and protected areas, may affect a possible genetic rescue of male horn length in hunted areas. Our simulations suggest that the effects of size‐selective harvest on horn length of male bighorn sheep can be attenuated within a metapopulation of harvested and protected populations, but only if there are substantial exchanges with little overlap with the hunting season. As expected, similar results can be obtained by lowering harvest pressure (Table S7). Importantly, our model predictions also suggest that if breeding migrations are substantial, intense selective harvest will impact protected populations, at the genetic, phenotypic, and demographic levels. The movement rate and the harvest risk of migrants from protected areas before they breed in exploited areas may condition the genetic divergence and variability between the two populations over time (Poisson et al., 2020; Tenhumberg et al., 2004). This is a key issue because if the source of genetic variance becomes depleted, then genetic rescue will be impossible even if males move between populations.

By varying the flow scale, we addressed the uncertainty in seasonal migration rate between harvested and protected populations at a large scale, whose variations in time and space can be affected by multiple factors such as distance and landscape connectivity (Chapman et al., 2011; Lassis et al., 2022a). We call for long‐term monitoring of wild populations within both exploited and protected areas to directly quantify temporal trends in genetic and phenotypic traits along with population dynamics. A better knowledge of the genetic compositions of and gene flow between harvested and protected populations is required to inform management to minimize harvest‐induced effects both inside and outside protected areas and favor genetic rescue (Pelletier et al., 2014).

Although much theoretical and laboratory work has advanced our understanding of mechanisms influencing dispersal and genetic rescue between spatially structured populations (Carlson et al., 2014; Holt, 1985; Holt & Gomulkiewicz, 1997; Johst et al., 1999; Parvinen et al., 2020), that knowledge is rarely applied in wildlife management and conservation, especially in terrestrial systems. The inclusion of marine no‐take reserves as part of landscape‐level management to restore and conserve marine biodiversity and enhance harvest in nearby exploited areas has expanded considerably in recent years (Di Franco et al., 2016; Sørdalen et al., 2020), and so has the use of modelling approaches to investigate their efficiency (Hutchings & Fraser, 2008; Hutchings & Kuparinen, 2020). The performance of protected areas in providing wildlife protection and ecosystem services at times conditions their financial support (Watson et al., 2014). Nevertheless, achieving these objectives depends on effective management that combines responsible harvest measures such as quotas and restrictions on the timing and location of harvest effort near protected areas (Di Franco et al., 2016; Hilborn & Kaiser, 2022; Melnychuk et al., 2021). In both marine and terrestrial systems, predictions from models of source‐sink dynamics could help evaluate the effectiveness of various management strategies to facilitate genetic rescue from protected areas and provide guidelines to conserve genetic and phenotypic variations at the metapopulation level, while maintaining harvest opportunities.

## CONFLICT OF INTEREST STATEMENT

None declared.

## Supporting information


Appendix S1.

## Data Availability

The R code used for the model construction and analyses is available on the Dryad Digital Repository doi: 10.5061/dryad.ksn02v78k.
