# Supplementary material for: Genetic rescue from protected areas is modulated by migration, hunting rate, and timing of harvest
Source: Evol Appl. 2023 May 12;16(6):1105–18. doi: 10.1111/eva.13554 (PMC10286230; doi:10.1111/eva.13554)
Supplement: Supplementary file 1 — Appendix S1. [file EVA-16-1105-s001.pdf]

Supplementary materials

S1. Supplementary figures

Figures S1: Model structure and parameters estimations

Figures S2: Effects of legal ram hunting rate without breeding migrations

Figures S3: Interplay between legal ram hunting rate and flow scale

Figures S4: Interplay between legal ram hunting rate and overlap of harvest with migrations

S2. Supplementary tables

S1. Supplementary figures

Figures S1: Model structure and parametrization

a)

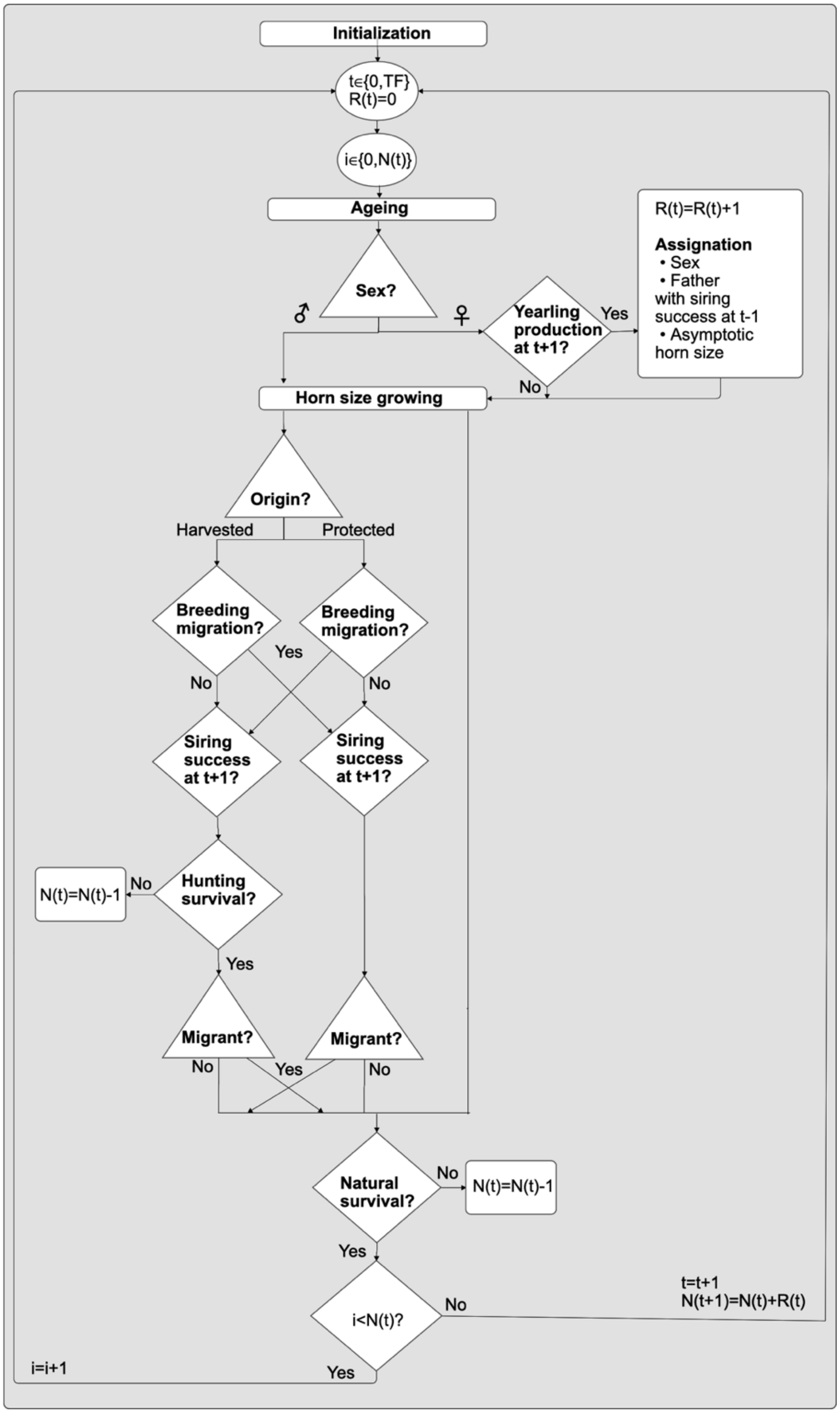

b)

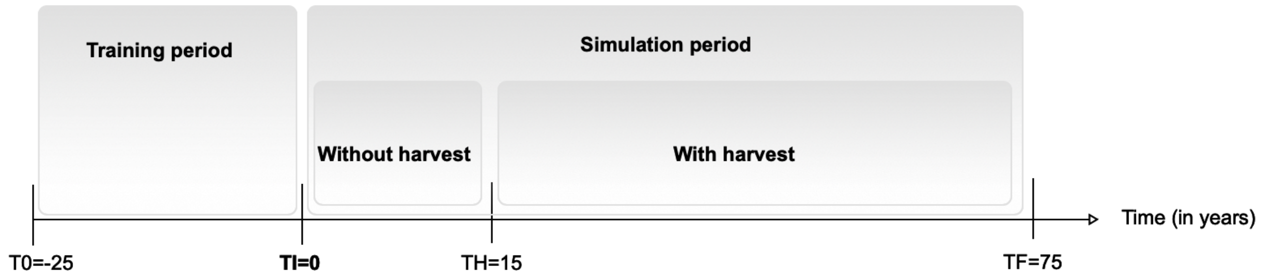

Figure S1.1: a) The simulation model with *t* as annual time step. T_F is the final simulation time, *R(t)* is the number of recruits at time *t*, *N(t)* is the total population size at time *t* and *i* is an index of a specific individual in the population. b) Simulation time scale where T_0, T_I, T_H and T_F designate the start of the training period, the simulation period, the onset of harvest and the end of simulation period, respectively.

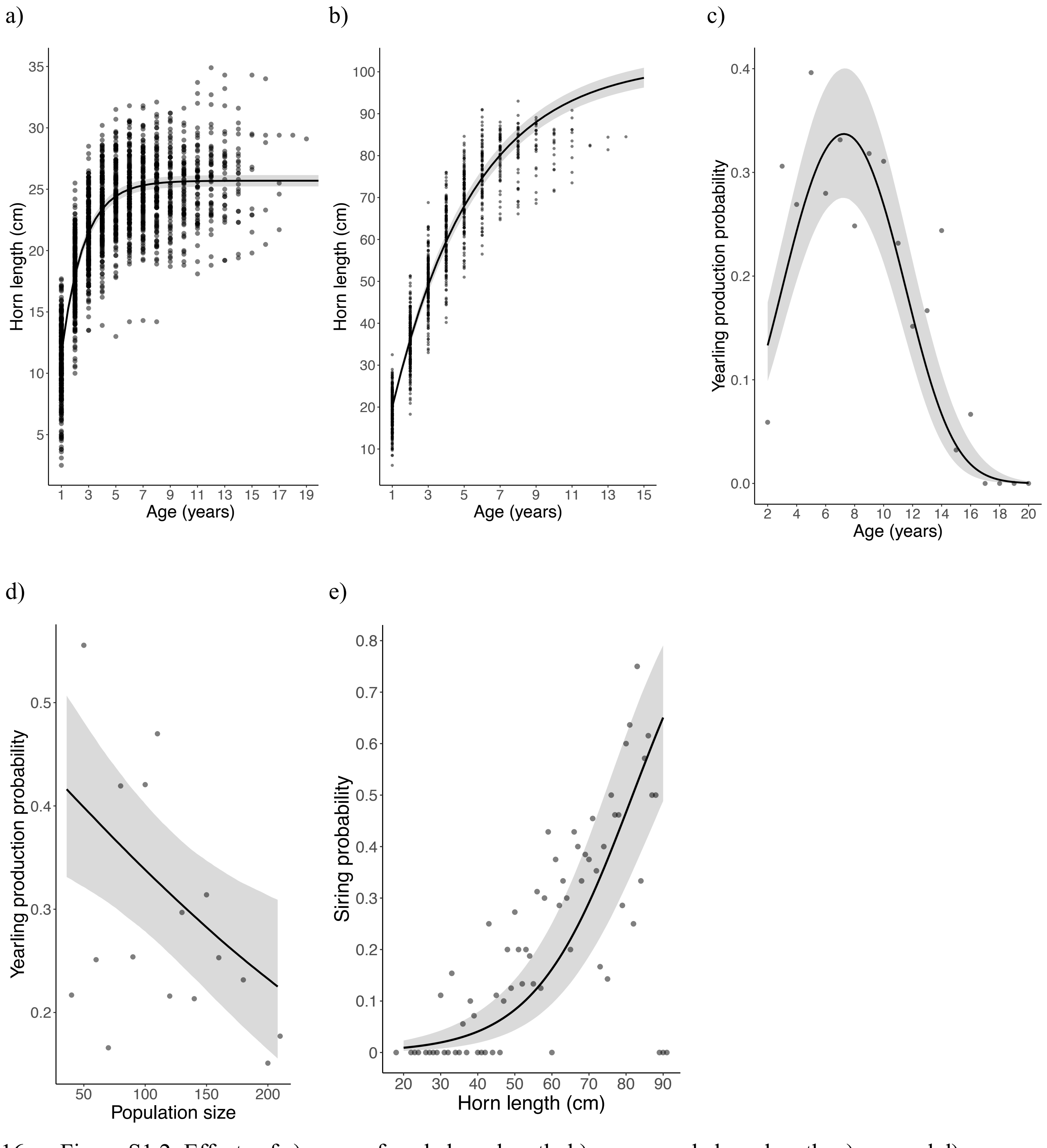

Figure S1.2: Effects of a) age on female horn length, b) age on male horn length, c) age and d) population size on yearling production probability of females, and e) horn length on male siring probability, Ram Mountain, Alberta, Canada. Regressions lines represent estimates from

Bayesian models with other non-focal variables set to their mean value (Table S2). Points represent mean sex- and age-specific observed horn length in panels a and b, observed proportions of female yearling production by age and population size in panels c and d, and observed proportions of male paternity according to horn length in panel e.

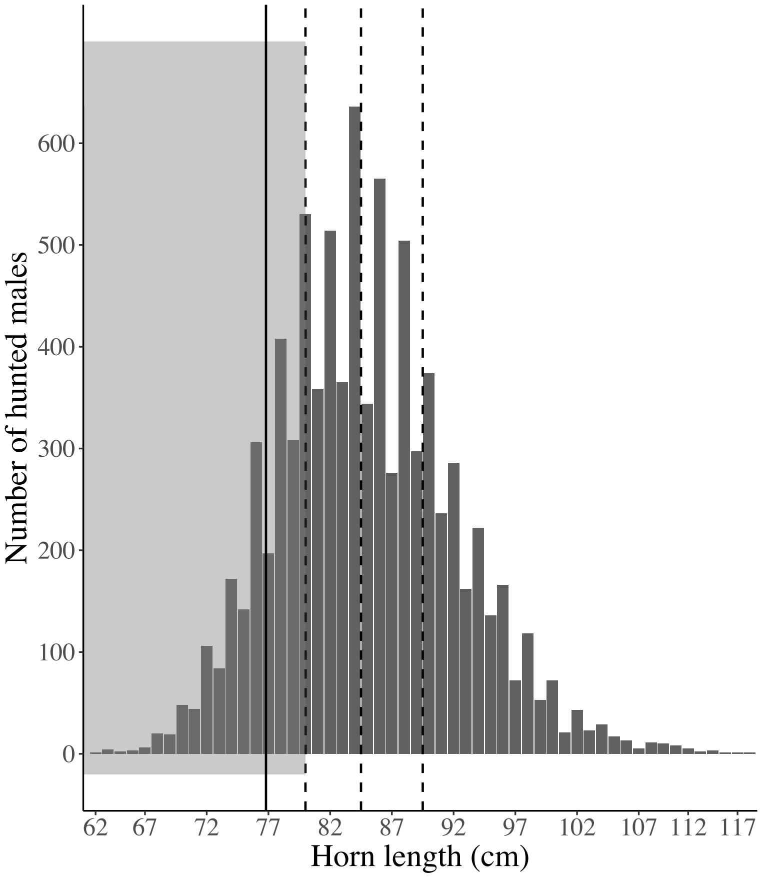

Figure S1.3: Histogram of horn length (longest total horn length along the outside curvature, in cm) of bighorn males shot under a 4/5-curl regulation in 1974-2019 in Alberta, Canada. The grey rectangle marks the lower portion of the distribution of horn length of hunted bighorn males, defined as the distribution below the 1st quartile. Value of minimum legal horn length y_{min} under the 4/5-curl rule was set to 76.8 cm, the mean of the lower distribution, marked with the solid vertical line. Left to right, the dashed vertical lines mark the 1st quartile, the median, and 3rd quartile of the overall distribution.

Figure S2: Effects of legal ram hunting rate without breeding migration

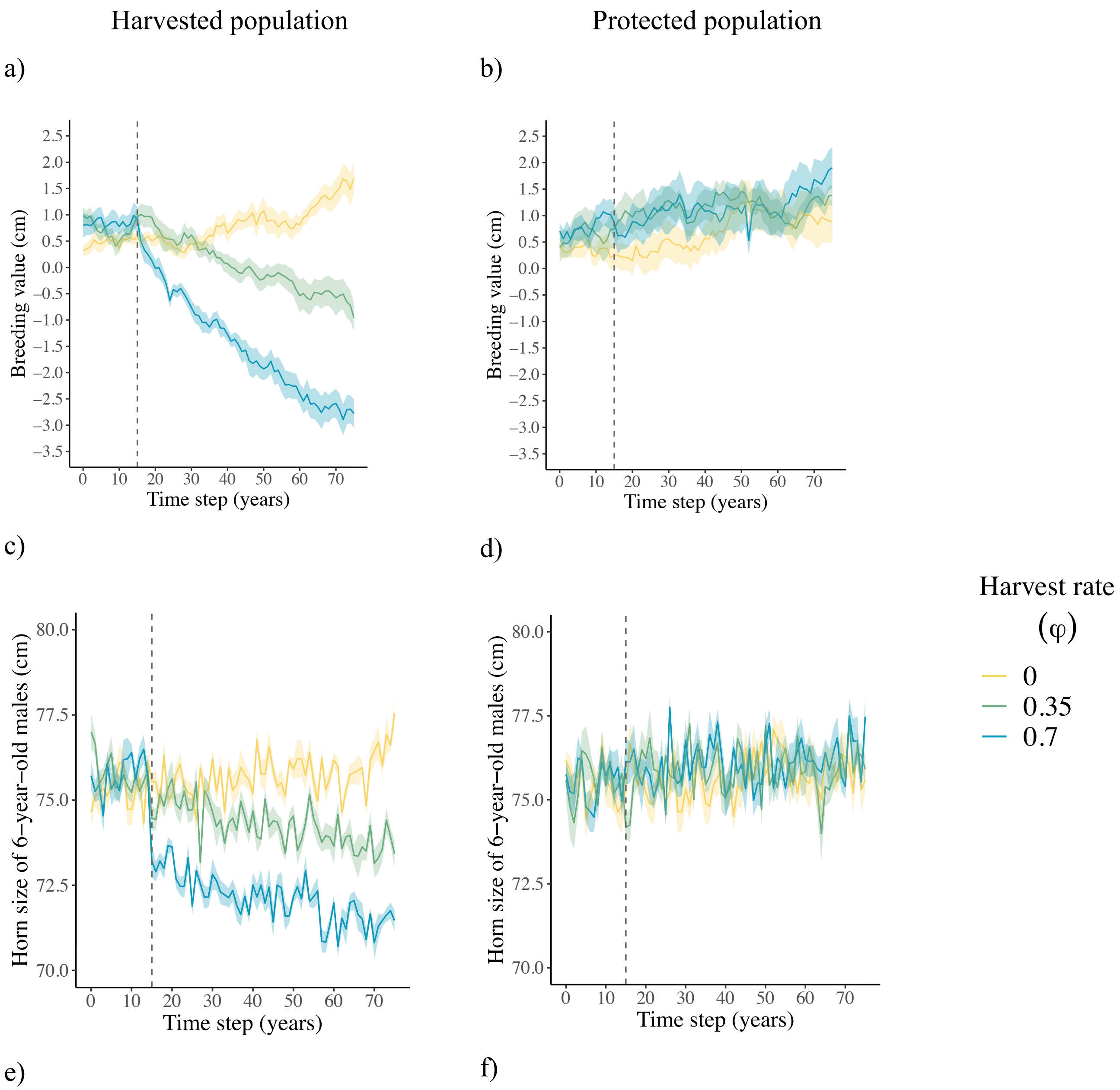

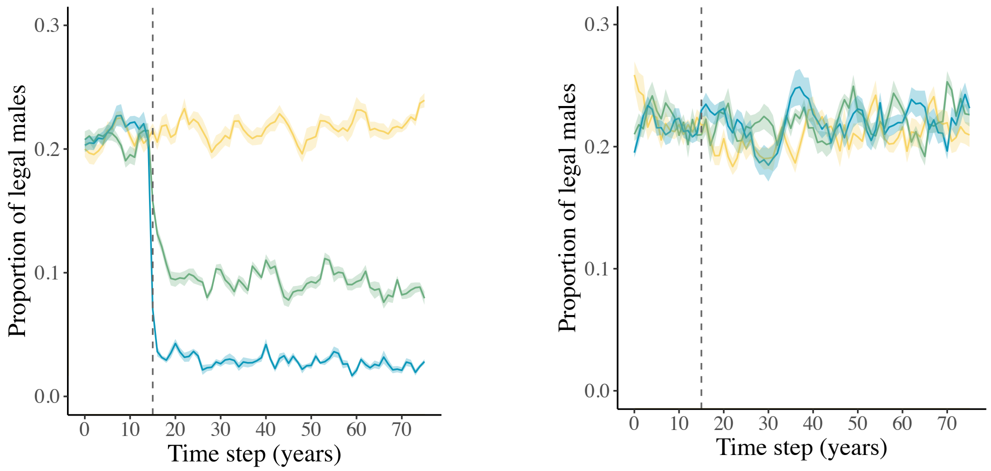

Figure S2.1: Effects of hunting rate (φ), without breeding migration (flow scale ε = 0.00) on the annual mean of male breeding value for male horn length (cm), 6-year-old males horn length (cm), and proportion of legal males in the harvested (a, c, e) and protected (b, d, f) populations. Lines and shades represent the averages and the associated 95% confidence intervals of all replicate runs. The vertical dashed line marks the start of annual harvest at T_H = 15 years.

Harvested population

Protected population

a)

b)

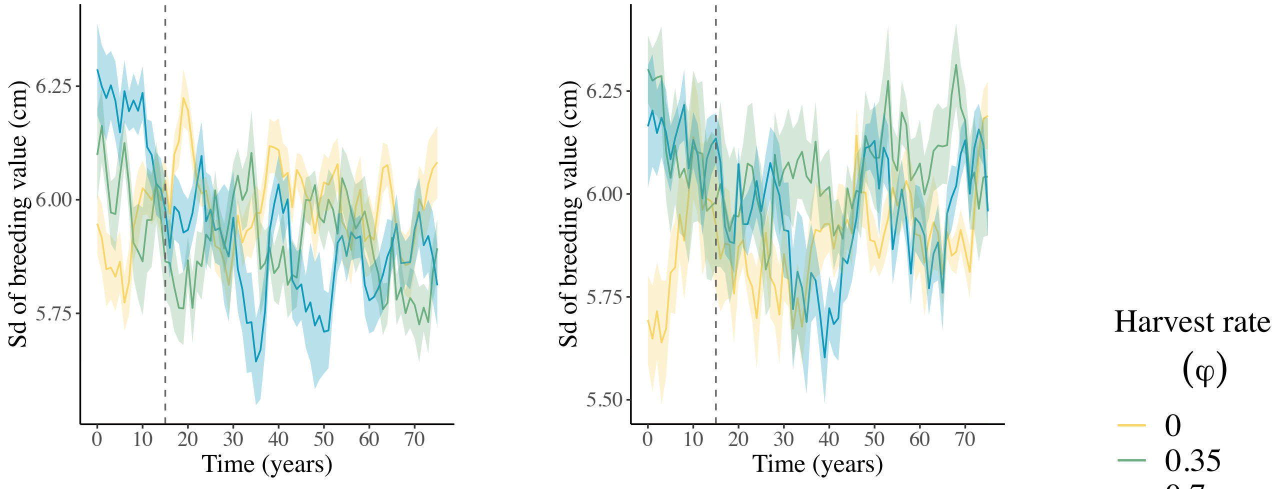

c)

d)

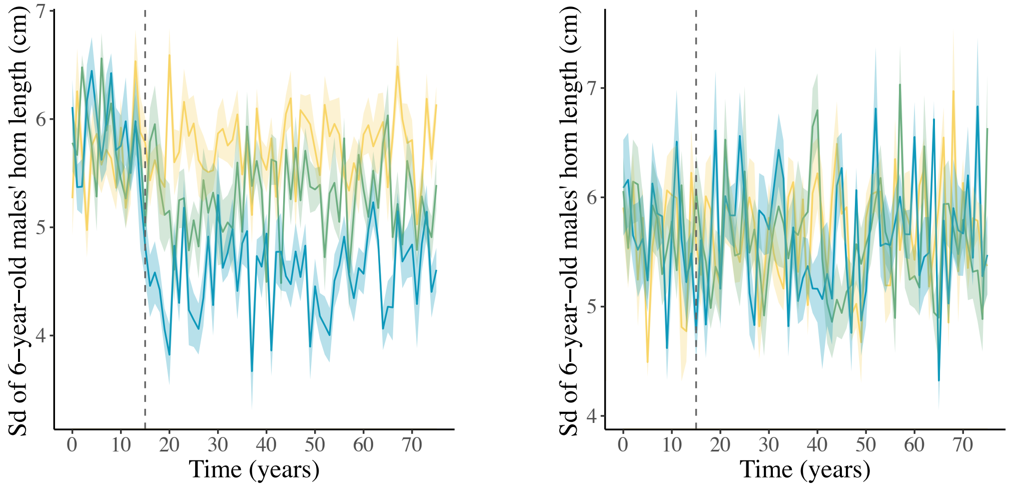

e) f)

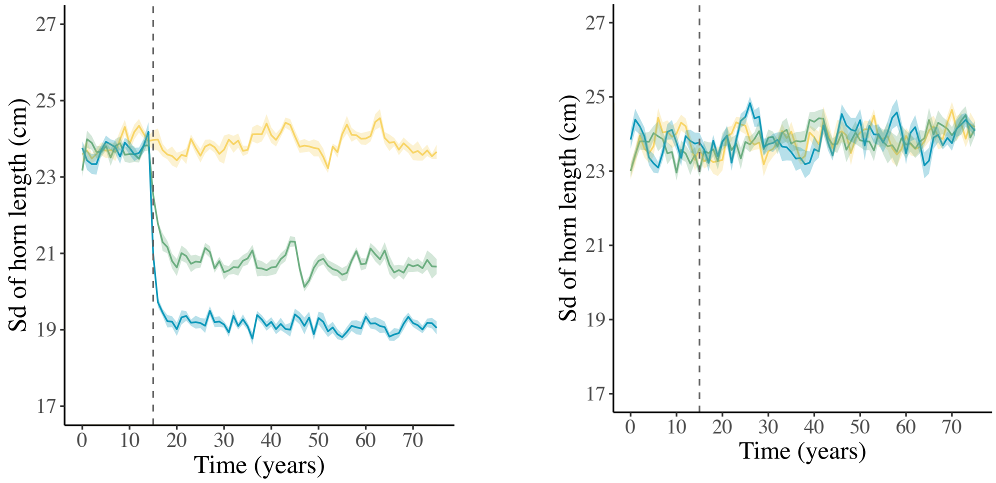

Figure S2.2: Effects of hunting rate (φ), without breeding migration (flow scale ε = 0.00) on the annual standard deviation of male breeding value for male horn length (cm), 6-year-old males horn length (cm), males horn length (cm) in the harvested (a, c, e) and protected (b, d, f) populations. Lines and shades represent the averages and the associated 95% confidence intervals of all replicate runs. The vertical dashed line marks the start of annual harvest at T_H = 15 years.

Figures S3: Interplay of legal ram hunting rate and flow scale

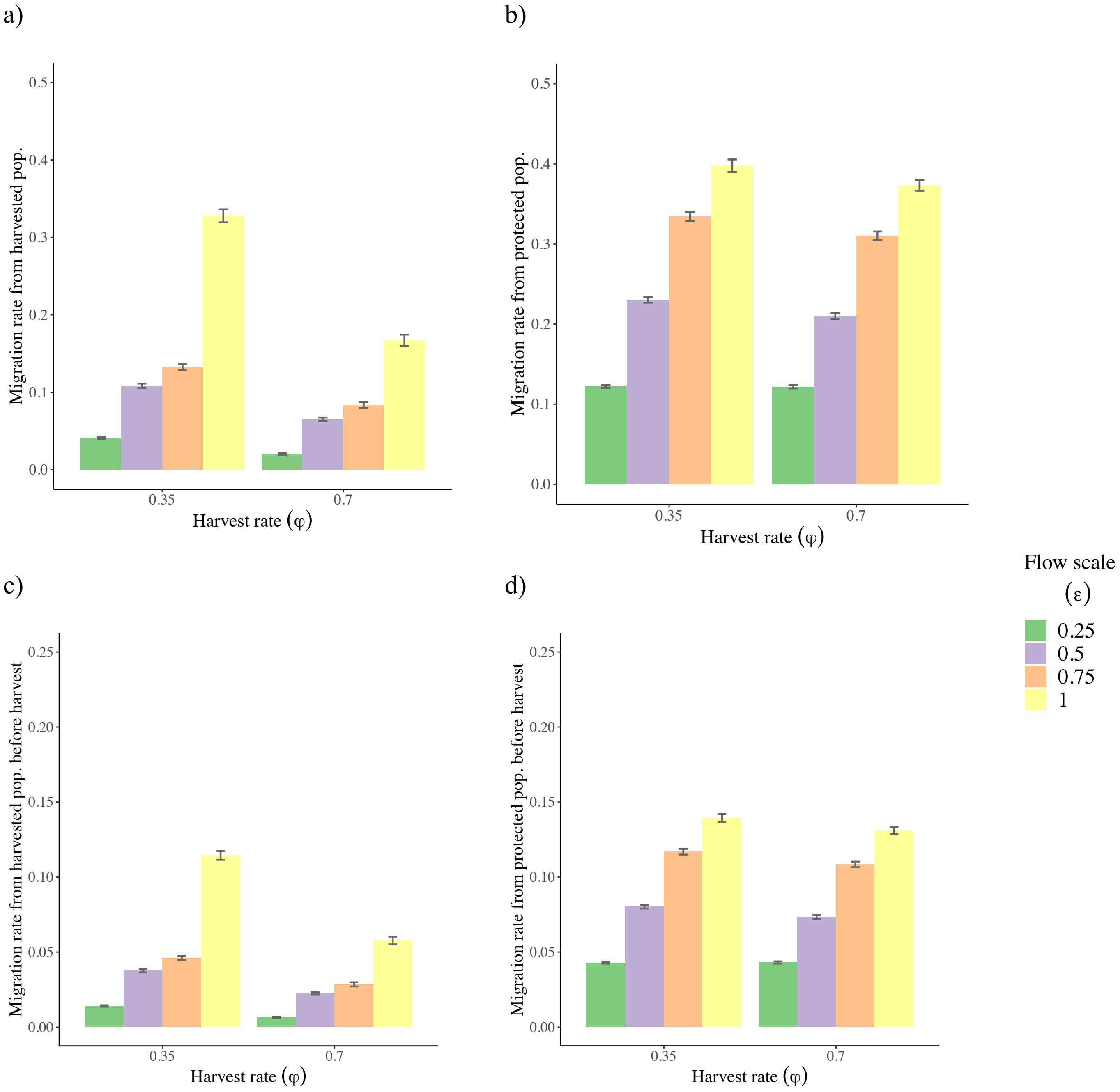

Figure S3.1: Yearly male breeding migration rates from harvested to protected population a) in total, (c) before harvest (i.e. proportions of protected males seasonally protected from hunting), and from protected to harvested populations b) in total, d) before harvest (i.e. proportions of harvested males seasonally exposed to hunting) according to hunting rate (φ, with timing of harvest overlap with migrations $\omega = 0.35$) and flow scale (ε) over 60 years of harvest (yearly

averages between T_H = 15 years and T_F = 75 years). Error bars represent 95% confidence intervals of yearly male breeding migration rates over all replicate runs.

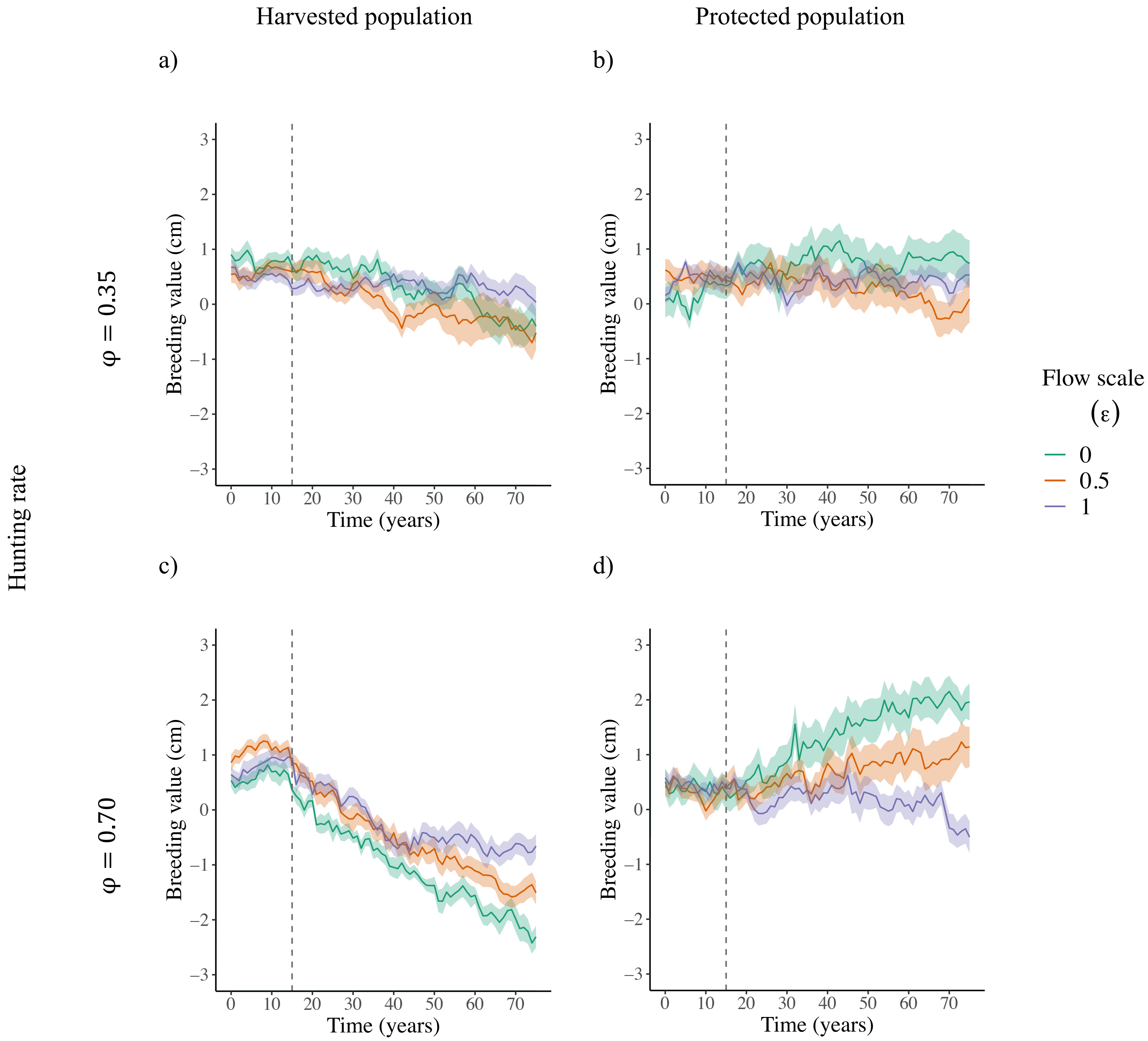

Figure S3.2: Effects of flow scale (ε) under hunting rates (φ = 0.35 or 0.70, with timing of harvest overlap with migrations ω = 0.35) on the annual mean breeding value for male horn length (cm) in harvested (a, c) and protected (b, d) populations. Lines and shades represent averages and associated 95% confidence intervals over all replicate runs. The vertical dashed line

marks the start of annual harvest at T_H = 15 years.

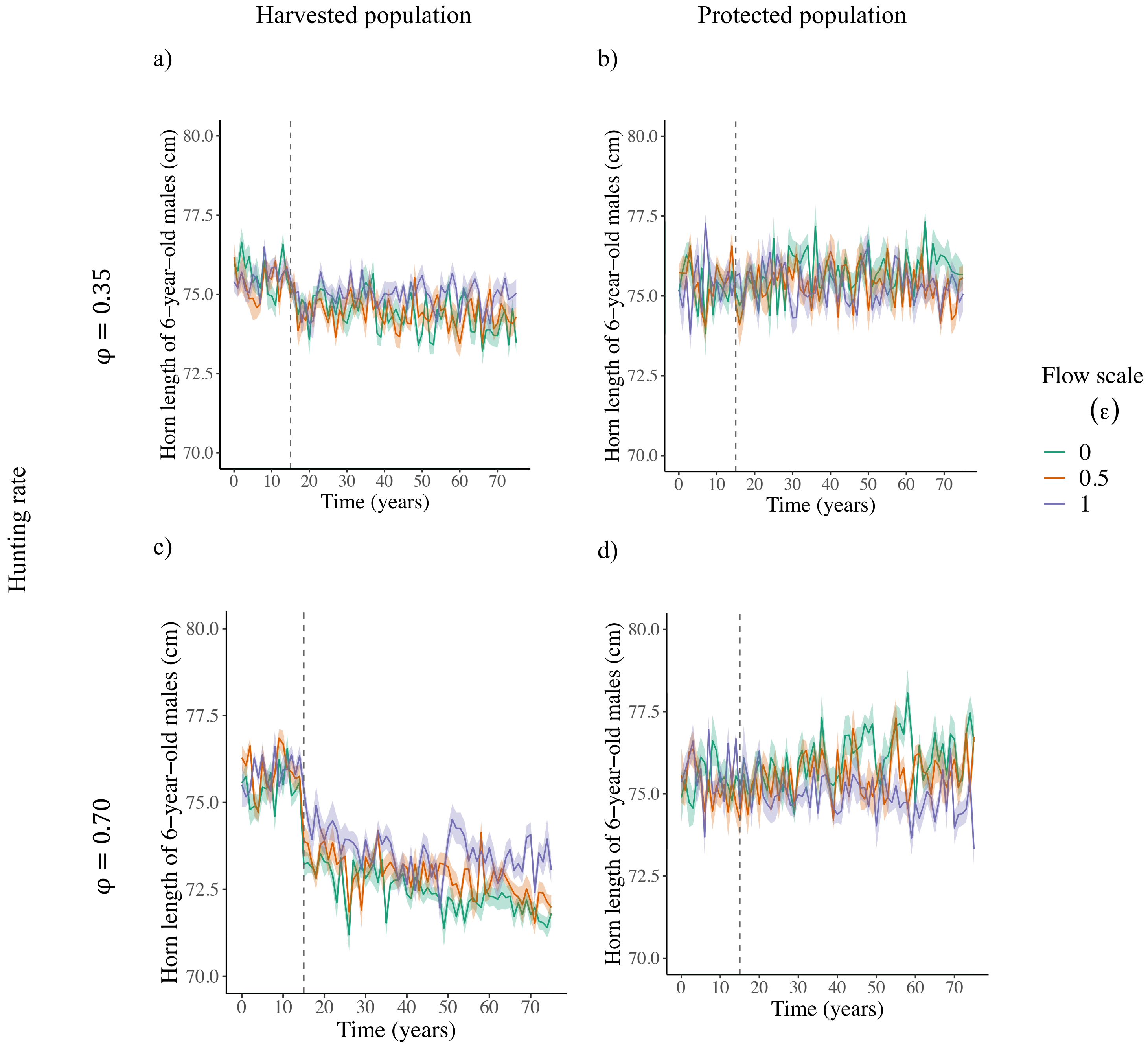

Figure S3.3: Effects of flow scale (ε) under different hunting rates (φ = 0.35 or 0.70, with timing of harvest overlap with migrations ω = 0.35) on the annual mean of 6-year-old males horn length (cm) in the harvested (a, c) and protected (b, d) populations. Lines and shades represent the averages and the associated 95% confidence intervals of all replicate runs. The vertical dashed line marks the start of annual harvest at T_H = 15 years.

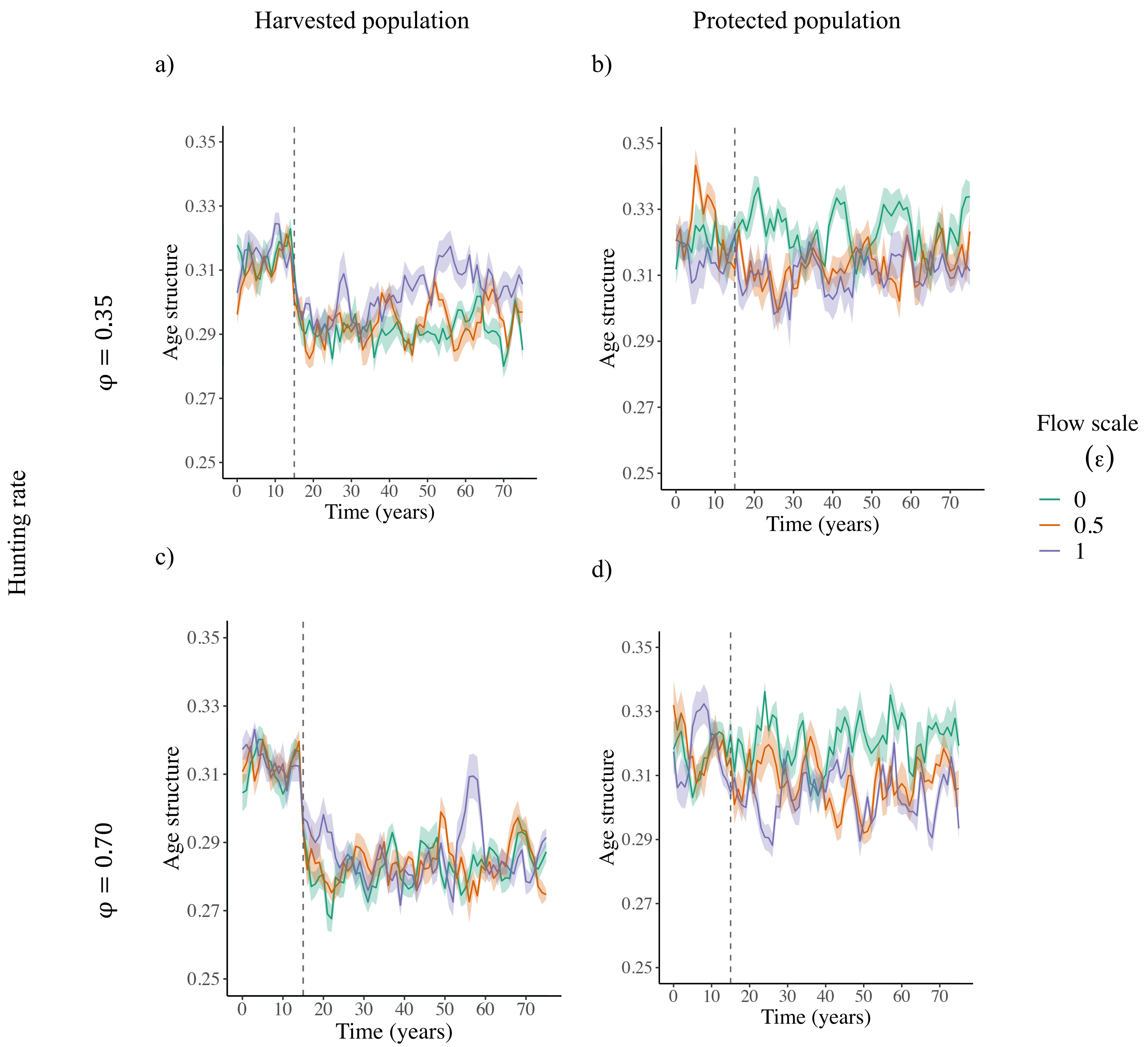

Figure S3.4: Effects of flow scale (ε) under different hunting rates ($\varphi = 0.35$ or 0.70, with timing of harvest overlap with migrations $\omega = 0.35$) on the annual age structure (proportion of 7-year-old and older individuals) in the harvested (a, c) and protected (b, d) populations. Lines and shades represent the averages and the associated 95% confidence intervals of all replicate runs. The vertical dashed line marks the start of annual harvest at $T_H = 15$ years.

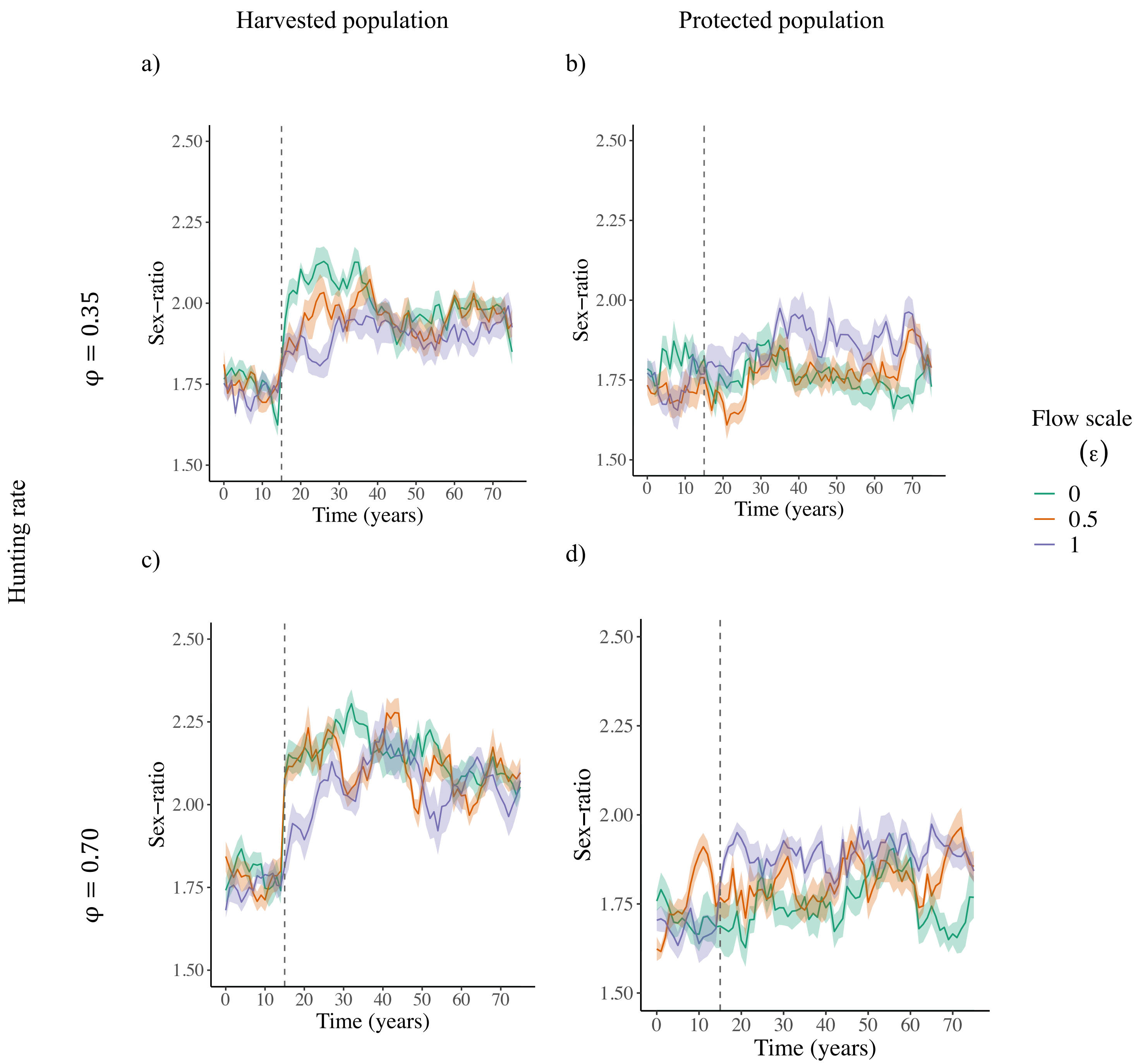

Figure S3.5: Effects of flow scale (ε) under different hunting rates ($\varphi = 0.35$ or 0.70, with timing of harvest overlap with migrations $\omega = 0.35$) on the annual female per male sex-ratio in the harvested (a, c) and protected (b, d) populations. Lines and shades represent the averages and the associated 95% confidence intervals of all replicate runs. The vertical dashed line marks the start of annual harvest at $T_H = 15$ years.

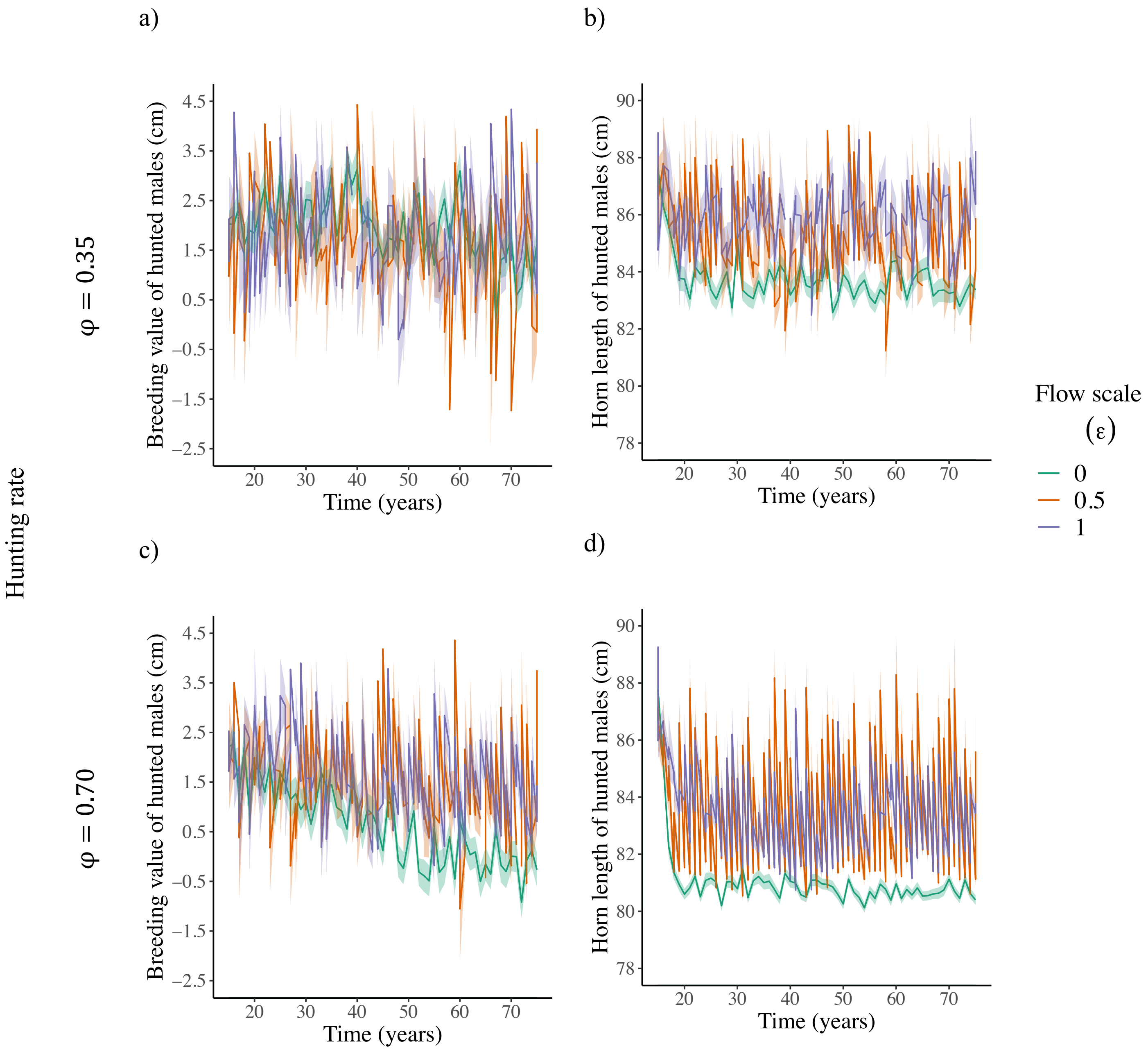

Figure S3.6: Effects (from harvest start at T_H = 15 years) of flow scale (ε) under different hunting rates ($\varphi = 0.35$ or 0.70, with timing of harvest overlap with migrations $\omega = 0.35$) on the annual mean breeding value for male horn length (cm) and horn length (cm) of hunted males of both origins (residents and migrants from the protected population). Lines and shades represent the averages and the associated 95% confidence interval between all replicate runs.

Figures S4: Interplay of legal ram hunting rate and overlap of harvest with migrations

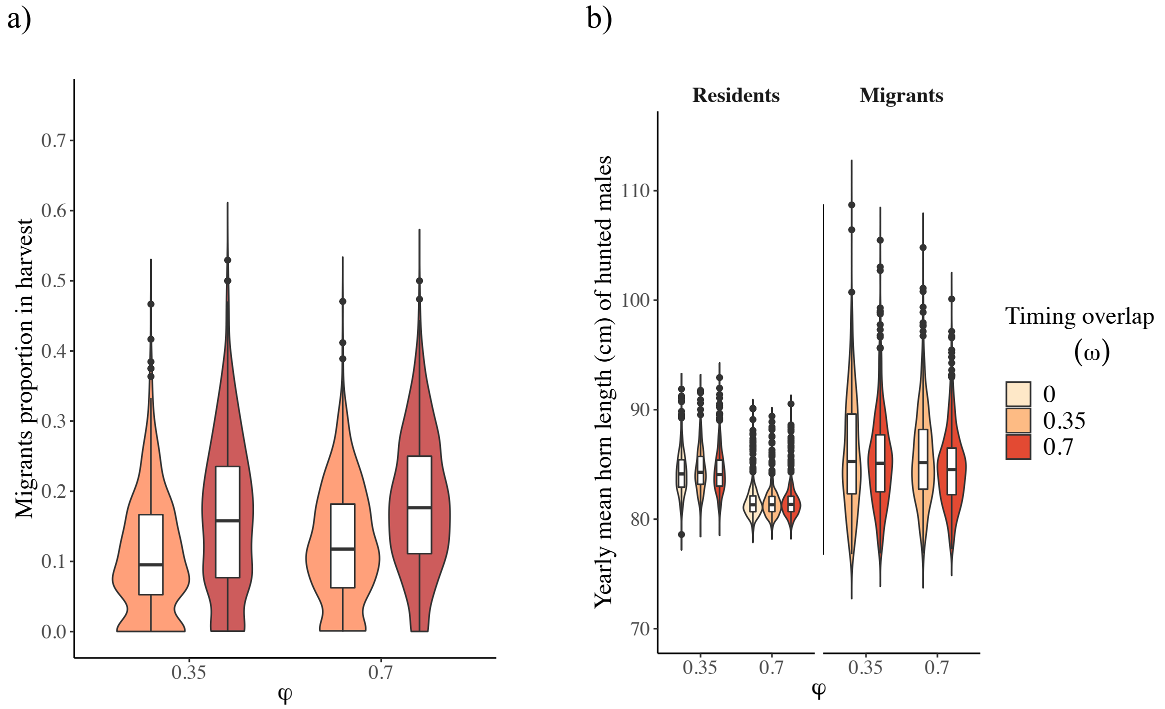

Figure S4.1: Combined effects of legal ram hunting rate (φ) and timing of harvest overlap with migrations (ω, with flow scale $\varepsilon = 0.50$) on a) the annual proportion of migrants males from protected population in the yearly harvest and b) the yearly mean horn length of harvested males (y_m) according to their origin (residents or migrants from the protected population) over 60 years of harvest (yearly averages between T_H = 15 years and T_F = 75 years). The black line in the box indicates the median, the lower and upper edges mark the 1st and 3rd quartiles, and the lower and upper whiskers extend respectively to the smallest and largest values no further than 1.5 times the inter-quartile range over all replicate runs. Violin shapes represent density estimates of the *y*-axis variables in each combination of values for φ and ω.

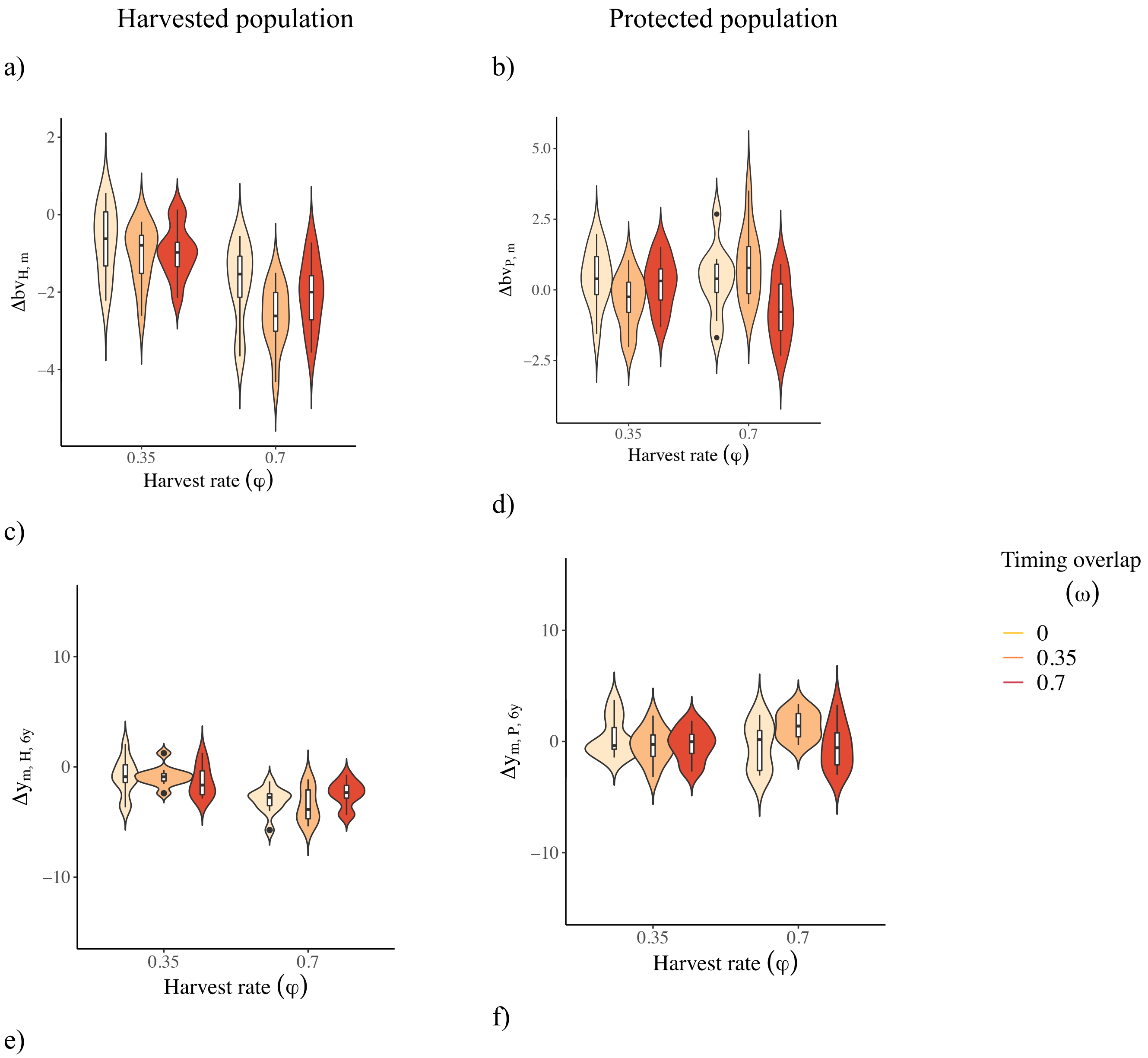
Harvested population
Protected population
a)
b)
c)
d)
e)
f)
$\Delta bv_{H,m}$
$\Delta bv_{P,m}$
$\Delta y_{m,H,6y}$
$\Delta y_{m,P,6y}$
Harvest rate (φ)
0.35
0.7
Timing overlap (ω)
0
0.35
0.7

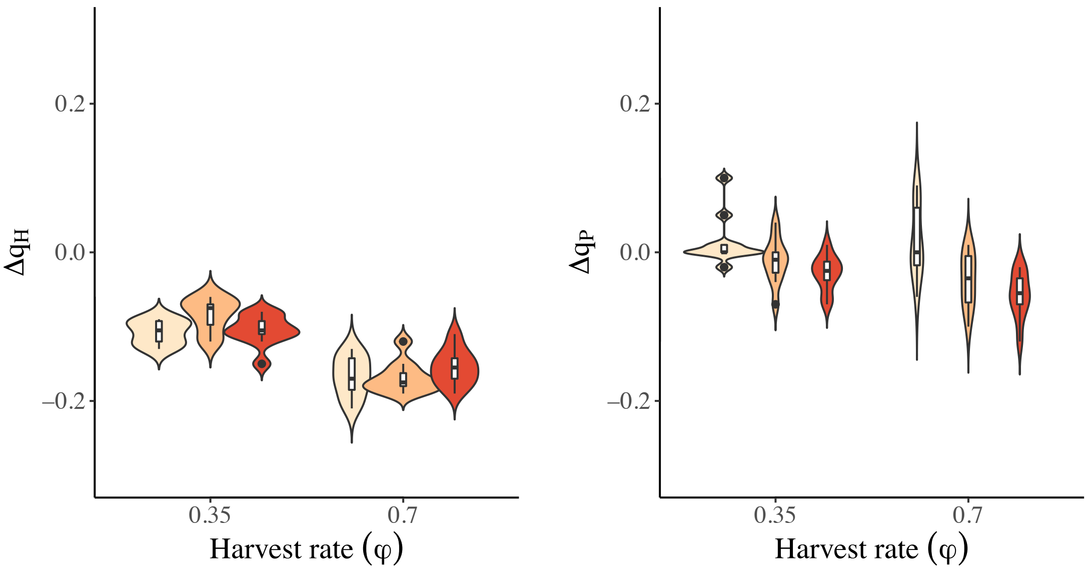

Figure S4.2: Combined effects of legal ram hunting rate (φ) and timing of harvest overlap with migrations (ω, with flow scale $\varepsilon = 0.50$) on the temporal trends over 60 years of harvest (differences between the average value before T_F = 75 years and before T_H = 15 years) in mean breeding value for male horn length (Δbv_m in cm), horn length of 6-year-old males ($\Delta y_{m,6y}$ in cm), and proportion of legal males (Δq) in the harvested (a, c, e) and protected (b, d, f) populations. The black line in the box indicates the median, the lower and upper edges mark the 1st and 3rd quartiles, and the lower and upper whiskers extend respectively to the smallest and largest values no further than 1.5 times the inter-quartile range over all replicate runs. Violin shapes represent density estimates of the y-axis variables in each combination of values for φ and ω.

S2. Supplementary tables

Table S1: Definition of model variables tracking populational and individual features in simulated populations of bighorn sheep.

Description	Symbol	Details
**Time**		
Time	t	in years
**Population features**		
Total population size	N	number of individuals
Protected population size	N_P	number of individuals
Harvested population size	N_H	number of individuals
**Individual features**		
Age	a	at start of time step
Sex	s	female (F) or male (M)
Population origin	o	protected (P) or harvested (H)
Asymptotic horn length	y_∞	cm
Horn length	y	cm
Breeding value for female horn length	x_f	cm
Breeding value for male horn length	x_m	cm

Table S2: Models fitted, sample sizes used (*F* for number of females/year and *M* for number of males/year), and final estimates with corresponding 95% confidence intervals used in sensitivity analysis for model parameters. Models were fitted using Bayesian generalized non-linear mixed models the "BRMS" library (Bürkner 2017) on long-term data from Ram Mountain bighorn population, 1975-2019, Alberta, Canada. In model formula, $(A|B)$ denotes variable *A* is random over the levels of factor *B*.

Description	Method	Model	Sample size	Coefficients [95% *CI*]	Figure

**Horn growth**	Gaussian family Identity link	$y^{*} \sim y_{\infty}(1 - e^{-\rho a})$<br>*y:* horn length (cm)<br>$y_{\infty} \sim 1 + (1\|Id + Cohort)$<br>$\rho \sim 1 + (1\|Id + Cohort)$<br>*a: age (year)*	*F: 2010*<br>*M: 1190*	$y_{\infty,f} = 25.69$ [25.23; 26.15]<br>$y_{\infty,m} = 102.51$[99.89; 105.25]<br>$\rho_f = 0.60$[0.57; 0.63]<br>$\rho_m = 0.22$[0.21; 0.23]	Fig. S1.2a<br>Fig. S1.2b
**Breeding**	Bernoulli family Logit link	$p_{recruit}^{**} \sim B(\theta)$<br>$p_{recruit}$: female probability to produce a yearling<br>$\theta \sim \theta_0 + \theta_1 a + \theta_2 a^2 + \theta_3 N + (1\|Id + Cohort)$<br>$a$: age (year)<br>$N^{***}$: population size	*F: 2112*	$\theta_0 = -2.96[-3.10; -1.91]$<br>$\theta_1 = 0.65[0.51; 0.80]$<br>$\theta_2 = -0.04\ [-0.05; -0.03]$<br>$\theta_3 = -0.01[-0.01; 0.00]$	Fig. S1.2c<br>Fig. S1.2d
	Bernoulli family Logit link	$p_{siring}^{****} \sim B(\gamma)$<br>$p_{siring}$: male siring probability<br>$\gamma \sim \gamma_0 + \gamma_1 y + (1\|Id + Cohort)$<br>*y*: horn length (cm)	*M: 688*	$\gamma_0 = -6.21[-7.60; -4.96]$<br>$\gamma_1 = 0.08\ [0.06; 0.09]$	Fig. S1.2e

* Last annual measure of longest total horn length along the outside curvature of all females and males, 1975-2019, Ram Mountain, Alberta, Canada.

**Annual reproductive status (production of lamb that survived to 1 year of age) of all females aged 1 year and older, 1975-2016, Ram Mountain, Alberta, Canada.

***Annual number of individuals aged 1 year and older of both sexes, 1975-2019, Ram Mountain, Alberta, Canada.

****Siring success of all males aged 2 years and older according to their longest total horn length along the outside curvature, in 1984-2017 (except 1993, year with no paternity assigned), Ram Mountain, Alberta, Canada.

Table S3: Mean estimates and corresponding standard errors or 95% confidence intervals derived from the literature and used in sensitivity analysis for model parameters values. In model formula, ($A|B$) denotes variable A is random over the levels of factor B.

Description	Model	Data (Source)	Coefficients $\pm SE$ / [95% *CI*]
**Offspring**	Animal model<br>$y \sim aI + bG + cE + R$<br>$+(1\|Id + Year + Cohort)$<br>y^*: horn length (cm)<br>I: individual features<br>A: additive genetic<br>E: environment<br>R: residuals<br>Id: individual identity<br>$\mathrm{V_P} = \mathrm{V_G} + \mathrm{V_E};\ \mathrm{h}^2 = \frac{\mathrm{V_G}}{\mathrm{V_P}}$<br>$h^2$: heritability<br>$V_P$: phenotypic variance<br>$V_G$: genetic variance<br>$V_E{}^{**}$: non-genetic variance	Ram Mountain bighorn population, 1973-2011, Alberta, Canada (Table 1, Pigeon et al. 2016)	$h_f^2 = 0.223\ [0.090;\ 0.446]$<br>$h_m^2 = 0.397\ [0.203;\ 0.534]$<br>$V_{G_{ff}} = 1.622$ [0.748; 3.963]<br>$V_{G_{fm}} = 5.345$ [1.928; 8.144]<br>$V_{G_{mm}} = 17.884$ [9.82; 25.881]<br>$V_{E_{ff}} = \frac{V_{G_{ff}}}{\mathrm{h_f^2}} - V_{G_{ff}}$<br>$V_{E_{mm}} = \frac{V_{G_{mm}}}{\mathrm{h_m^2}} - V_{G_{mm}}$
**Migration**	Descriptive statistics<br>$\mathrm{p_{permanent\ migration}} \sim \mathrm{B}(\varepsilon_0)$	Ram Mountain bighorn population, 1975-2019, Alberta, Canada (Unpublished data)	$\varepsilon_0 \approx 0.000$

	Bayesian generalized non-linear mixed model<br>$p_{breeding\ migration} \sim B(\lambda)$<br>$\lambda \sim \lambda_0 + \lambda_1 a + \lambda_2 a^2 + \lambda_3 sr + \lambda_4 N_m + (1 \vert Id + Year)$<br>$a$: age (year)<br>$sr$: sex ratio (female/male)<br>N_m: number of males	Sheep River bighorn population, 2000-2005, Alberta, Canada (Table 1, Lassis et al. in press)	$\lambda_0 = -10.79[-30.74; 8.86]$<br>$\lambda_1 = 9.02[4.00; 15.35]$<br>$\lambda_2 = -0.61\ [-4.58;\ 3.19]$<br>$\lambda_3 = -5.31[-11.37; 0.39]$<br>$\lambda_4 = 0.49\ [0.10; 0.92]$
**Natural mortality**	Capture–mark–resighting model<br>$p_{survival} \sim B(\mu)$<br>$\mu \sim \mu_0 + \mu_1(a-8) + \mu_2(a-8)^2$<br>$a$: age (year)	Ram Mountain bighorn population, 1975-1997, Alberta, Canada (Table 6, Loison et al. 1999)	$\mu_{0,f} = 2.059\ \pm 0.143$<br>$\mu_{1,f} = -0.172\ \pm 0.028$<br>$\mu_{2,f} = -0.001\ \pm 0.006$<br>$\mu_{0,m} = 1.028\ \pm 0.183$<br>$\mu_{1,m} = -0.165\ \pm 0.096$<br>$\mu_{2,m} = 0.0006 \pm 0.017$

* Longest total horn length along the outside curvature

** Non-genetic variance encompasses environmental and residual variances

Table S4: Simulation time and initial population features used as initial conditions for all simulation runs. Population sizes were set to 1/10 of density estimates within both protected and harvested populations in Alberta (Jorgenson 2008).

Description	Symbol	Values
**Time**		
Start of harvest selection	T_H	15 years
Final simulation time	T_F	75 years
**Initial population features**		
Total population size	N_0	1 100 individuals
Protected population size	$N_{0,P}$	450 individuals

Harvested population size	$N_{0,H}$	650 individuals

Table S5: Explored values for model parameters.

Description	Symbol	Equation	Parameter value	Description
**Migration**				
Flow scale	ε	Eq. 7	0.00, 0.50, 1.00	Explored levels of flow between both populations
**Harvest**				
Hunting rate	φ	Eq. 9	0.00, 0.35, 0.70	Explored hunting rates of legal males (Festa-Bianchet and Mysterud 2018)
Timing overlap	ω	-	0.00, 0.35, 0.70	Explored degree of overlap between hunting season and breeding migrations (ie. percentage of migrations executed while hunting is applied, Lassis et al. in press)

Table S6: Values of explored model parameters and associated simulation scenarios.

		Model parameters								
		Migration			Harvest					
		Flow scale (ε)			Legal ram hunting rate (φ)			Timing overlap with migrations (ω)		
Hypothesis	Scenario	0.00	0.50	1.00	0.00	0.35	0.70	0.00	0.35	0.70
	1	✓			✓			✓		
Control	2	✓				✓		✓		

	3	✓					✓	✓		
H1. Effects of hunting rate	1		✓		✓				✓	
	2		✓			✓			✓	
	3		✓				✓		✓	
H2. Interplay of hunting rate and flow scale	1	✓			✓				✓	
	2	✓				✓			✓	
	3	✓					✓		✓	
	4		✓		✓				✓	
	5		✓			✓			✓	
	6		✓				✓		✓	
	7			✓	✓				✓	
	8			✓		✓			✓	
	9			✓			✓		✓	
H3. Interplay of hunting rate and timing	1		✓		✓			✓		
	2		✓			✓		✓		
	3		✓				✓	✓		
	4		✓		✓				✓	
	5		✓			✓			✓	
	6		✓				✓		✓	
	7		✓		✓					✓
	8		✓			✓				✓
	9		✓				✓			✓

Table S7: Effects of hunting rate (φ), flow scale (ε) and timing of harvest overlap with migrations (ω) on temporal trends for breeding value of male horn length within harvested population over 60 years of harvest. Negative values mark temporal declines over 60 years, computed as the differences between the average final value at T_F=75 years and the average

value before harvests start at T_H=15 years. Mean differences over 10 replicate runs of each scenario are presented, with minimum and maximum differences in brackets.

Parameter value

	φ	0.00	0.35			0.70		
ε	ω	0.00	0.00	0.35	0.70	0.00	0.35	0.70
0.00		0.49	-1.33	-	-	-3.01	-	-
		[-0.06, 0.87]	[-2.88, 0.33]			[-4.31, -2.24]		
0.50		1.07	-0.66	-1.03	-0.95	-1.76	-2.62	-2.03
		[-0.65, 3.62]	[-2.19, -0.58]	[-2.60, 0.12]	[-2.12, 0.18]	[-3.67, -0.52]	[-4.32, -1.50]	[-3.57, -0.67]
1.00		1.01	-0.38	-0.60	-0.57	-1.04	-1.32	-1.72
		[-0.61, 2.21]	[-1.64, 0.89]	[-2.43, 1.06]	[-1.95, 1.15]	[-2.34, 0.39]	[-3.02, -0.60]	[-3.31, -0.44]

Table S8: Sensitivity analysis for model parameters. Decline values, $D_{sens,\pm5\%}$, are the mean temporal declines in breeding value of male horn length within harvested population over 60 years of harvest obtained between 50 simulation replicate runs with each focus parameter varying by ±5%. The first and third quartile of obtained declines over the 50 replicate runs are between brackets. Sensitivity values, $S_{\pm5\%}$* are the ratios between the observed change in temporal declines in breeding value of male horn length and the perturbation in a parameter p, so that large values indicate large sensitivity of the model to a change in this parameter (Jørgensen 1986). Within a sensitivity test, all 50 replicate runs started with the same initial harvested and protected populations and were executed with the same parametrization.

Parameter values	Perturbation at -5%		Perturbation at +5%	
	$D_{sens,-5\%}$ (cm)	$S_{-5\%}$	$D_{sens,+5\%}$ (cm)	$S_{+5\%}$
**Horn growth**				
ρ_f	0.99 [0.47, 1.67]	1.14	1.21 [0.58, 2.06]	3.05

ρ_m	1.06 [0.32, 1.70]	0.19	0.90 [0.33, 1.54]	2.86
$y_{\infty,f}$**	0.82 [0.17, 1.46]	4.38	0.79 [0.22, 1.40]	4.95
$y_{\infty,m}$**	0.85 [0.34, 1.48]	3.81	0.62 [0.12, 1.21]	8.19
**Breeding**				
γ_0	0.83 [0.31, 1.30]	4.19	1.31 [0.83, 1.82]	4.95
γ_1	1.29 [0.76, 1.80]	4.57	0.72 [0.20, 1.27]	6.29
**Offspring**				
$V_{G_{ff}}$	1.06 [0.40, 1.75]	0.19	1.15 [0.57, 1.71]	1.90
$V_{G_{fm}}$	1.25 [0.61, 1.92]	3.81	0.92 [0.24, 1.25]	2.48
$V_{G_{mm}}$	1.01 [0.56, 1.59]	0.76	1.10 [0.51, 1.59]	0.95
h_f^2	1.04 [0.66, 1.33]	0.19	1.00 [0.44, 1.57]	0.95
h_m^2	1.00 [0.38, 1.69]	0.95	0.86 [0.22, 1.62]	3.62
**Harvest**				
y_{min}	1.02 [0.50, 2.16]	0.57	0.93 [0.37, 1.56]	2.29

* Sensitivity values were computed as follows: $S_{\pm 5\%} = (\frac{D_{ref} - D_{sens,\pm 5\%}}{D_{ref}})/(\frac{p \pm 0.05 \times p}{p})$.

**All simulation runs started with the same initial harvested and protected populations, except when testing model sensitivity to changes in sex-specific asymptotic horn length, $y_{\infty,f}$ and $y_{\infty,m}$, that were used as input parameters to create the initial populations.
